# The Intensity of IUGR-Induced Transcriptome Deregulations Is Inversely Correlated with the Onset of Organ Function in a Rat Model

**DOI:** 10.1371/journal.pone.0021222

**Published:** 2011-06-22

**Authors:** Daniel Vaiman, Géraldine Gascoin-Lachambre, Farid Boubred, Françoise Mondon, Jean-Marc Feuerstein, Isabelle Ligi, Isabelle Grandvuillemin, Sandrine Barbaux, Eric Ghigo, Vincent Achard, Umberto Simeoni, Christophe Buffat

**Affiliations:** 1 Institut National de la Santé et de la Recherche Médicale (INSERM), Institut Cochin, Paris, France; 2 Université Paris Descartes, Paris, France; 3 CNRS, UMRS8104, Paris, France; 4 Division of Neonatology, Hôpital la Conception, Assistance Publique-Hôpitaux de Marseille, Marseille, France; 5 INSERM UMR608, Faculté de Pharmacie, Université de la Méditerranée, Marseille, France; 6 Centre National de la Recherche Scientifique, URMITE, Université de la Méditerranée, Unité Mixte de Recherche 6236, Marseille, France; 7 Laboratory of Reproductive Biology, Hôpital la Conception, Assistance Publique-Hôpitaux de Marseille, Marseille, France; 8 INSERM UMR 626, Faculté de Médecine, Aix-Marseille Université, Marseille, France; 9 Laboratoire de Biologie Moléculaire, Hôpital la Conception, Assistance Publique-Hôpitaux de Marseille, Marseille, France; Hôpital Robert Debré, France

## Abstract

A low-protein diet applied during pregnancy in the rat results in intrauterine growth restricted (IUGR) fetuses. In humans, IUGR is associated with increased perinatal morbidity, higher incidence of neuro-developmental defects and increased risk of adult metabolic anomalies, such as diabetes and cardiovascular disease. Development and function of many organs are affected by environmental conditions such as those inducing fetal and early postnatal growth restriction. This phenomenon, termed “fetal programming” has been studied unconnectedly in some organs, but very few studies (if any) have investigated at the same time several organs, on a more comparative basis. However, it is quite probable that IUGR affects differentially most organ systems, with possible persistent changes in gene expression. In this study we address transcriptional alterations induced by IUGR in a multi-organ perspective, by systematic analysis of 20-days rat fetuses. We show that (1) expressional alterations are apparently stronger in organs functioning late in foetal or postnatal life than in organs that are functioning early (2) hierarchical classification of the deregulations put together kidney and placenta in one cluster, liver, lungs and heart in another; (3) the epigenetic machinery is set up especially in the placenta, while its alterations are rather mild in other organs; (4) the genes appear deregulated in chromosome clusters; (5) the altered expression cascades varies from organ to organ, with noticeably a very significant modification of the complement and coagulation cascades in the kidney; (6) we found a significant increase in TF binding site for HNF4 proteins specifically for liver genes that are down-regulated in IUGR, suggesting that this decrease is achieved through the action of HNF transcription factors, that are themselves transcriptionnally induced in the liver by IUGR (x 1.84 fold). Altogether, our study suggests that a combination of tissue-specific mechanisms contributes to bring about tissue-driven modifications of gene cascades. The question of these cascades being activated to adapt the organ to harsh environmental condition, or as an endpoint consequence is still raised.

## Introduction

The period of intrauterine growth and development is one of the most vulnerable periods in the human life cycle. The weight of the infant at birth is a powerful predictor of infant growth and survival, and is dependent on maternal health and nutrition during pregnancy. Low birth weight leads to an impaired growth of the infant with its attendant risks of a higher mortality rate, increased morbidity [1], impaired mental development [2] and the risk of chronic adult disease [3]. Intra-uterine growth restriction (IUGR) is defined by a failure of the fetus to reach his/her normal (or genetically defined) growth potential [4]. In a clinical sense, it is therefore detectable by morphometric analysis of ultrasonographic scans followed by the conversion of length measures (femur, radius) to an estimation of weight [5]. The notion of SGA (Small for Gestational Age) is defined by a birth weight below the 10^th^ percentile at a given gestational age; it is actually currently used as an approximation for IUGR; however, it mainly refers to measurements carried out at birth, rather than to a dynamic measure involving an *in utero* rupture of the growth curve. IUGR is a very important health problem with a prevalence estimated at ∼10% in the general population [6]. It leads to ∼10% of the overall perinatal mortality [7]. IUGR, especially when it is not associated to vascular causes, obviously embraces a very heterogeneous spectrum of diseases in humans, hence the great utility of animal models to understand the physiopathology of this disorder [8], [9], [10]. In rats, a low birth weight can be induced in pups when dams are fed a isocaloric low protein diet (half to one third of the normal protein content, according to the studies, i.e. 8–10% versus 18–22%) during pregnancy [11]. This model has been used in the present study, since it is probably one of the most commonly used, and is known for decades to induce multiple organ alterations [12], [13], [14]. In this case, it has been shown that the offspring may retain anatomical and functional defects at adulthood in different organs [15]. Other studies have shown that the number of renal glomeruli is reduced in these pups [16] and remains low when a normal diet is given after birth. Anatomical and physiological variables in the offspring brain are also altered by this low protein diet, with a reduced number of dendrites, a lower sensory cortico-cortical and thalamo-cortical potentials, an elevated level of biogenic amines and alterations of tryptophan metabolism [17]. Hence, the low protein diet modifies drastically the metabolic environment of the fetus. Another interest of this model is that it reflects actual situations in some human cultures or harsh socio-economic situations. Today, the mechanisms inducing the various defects are imperfectly known, although they could be basically caused by general defects in vascular architecture [18], reducing the placental nutrient transfer [19], or targets of placental hormones [20].

Previously, we have reported specific transcriptome alterations in the placenta and in the kidney in a rat model of IUGR induced by a low-protein diet of the pregnant females [10], [21]. In the present study, we carried out on the same animal material a thorough transcriptional analysis including three additional organs: heart, liver and lungs. We showed that each organ (placenta, kidney, lung, liver and heart) displays a specific answer (both in intensity and in nature) to the environmental injury imposed to the pregnant rats. In addition, we pinpoint organ-specific differences for genes involved in epigenetic regulation of expression (DNA methyl-transferases, Histone deacetylases, Histone methyl and acetyl transferases, Chromodomain and Bromodomain protein-encoding) and imprinted genes. We also carried out a per-chromosome analysis which reveals clusters of genes that are more specifically modified by IUGR.

Our major conclusions sustain the existence of strong tissue-specific differences in the intensity of transcriptional alterations. The analysis of modified genes shows that various organ-dependent gene pathways are altered and that in term of gene expression modifications, kidney and placenta are clustered versus the three other organs (liver, lung, heart). Genes modulating the epigenetic status of the chromatin are systematically activated in the placenta, while the situation is more contrasted in other organs. We show that the intensity of the response is linked to the moment when each organ becomes functional, i.e. the earliest being the least affected. We propose that since stable epigenetic modifications are less consequential in the placenta, which is transient, they are tolerated if they can improve the response of this organ to deleterious environmental conditions (i.e. shortage of nutrients, and especially proteins). In the other organs, our results suggest that transcriptional alterations, as important as they can be, rely mainly on transient, transcription factor-dependent deregulations. This does not discard the possibility of targeted epigenetic alterations as have been evidenced for IGF1 in the liver [22].

## Materials and Methods

### Animals

The ethical committee for animal research at Marseille Medical University approved the experiments. The animals were kept under standard conditions according to the recommendations for the use of animals in experimental designs and according to the “3R” rules. The authorization number for the experiments is: D13–491. The agreement of the animal facility is registered under the number F13-055-5. Female virgin Sprague Dawley rats (Charles Rivers, l'Abresle, France) weighing 225–250 g were used. After mating, the presence of sperm in the vaginal smear the following morning was designated as day 1 post-coitum. The rats were fed either a 22% (w/w) casein (control) or a 9% (w/w) casein (LP) diet throughout gestation, as described previously [21]. Both diets were isocaloric, because the protein deficiency in the LP diet was compensated by the addition of carbohydrates. During pregnancy animals were weighed and food intake was recorded daily. The pregnant rats about 100 days old, at their first gestation, were euthanized following anesthesia with halothane-nitrous oxide at gestation day 20 (20–21 days post coitum) and placentas and fetal tissues were rapidly removed, weighed and frozen in liquid nitrogen and stored at −80°C for later RNAs expression analysis. The choice of sacrificing the females at 20 days was dictated by the necessity to be sure to obtain the placentas, since the normal gestation is 21–23 days. A total of 77 and 67 fetuses in 6 and 5 litters, respectively, were weighed for the LP diet and the control group, respectively. Alterations in relative and absolute fetal organ weight have been already published, see Table S3 [21]. Absolute organ weights were in general reduced by ∼20%, but the placental weight was reduced of only 12%. Relative to body weight, liver, heart and kidneys were still significantly reduced in weight. Technically speaking, there is no official growth curve for rats, then for maximizing the effects detected in the microarray analysis, the most extreme organs in term of weights were chosen in the control and the treated group. This way, we hypothesize that the most drastic cases of IUGR have been considered in the study.

### RNA preparation, cDNA synthesis, and microarray hybridizations

RNA isolation was performed according to standard protocols using Trizol reagent and controlled (precise integrity checks and sample quantitation) by Agilent bioanalyser 2100. Ten micrograms total RNA from a pool of more than 30 fetal tissues from LP group and a pool of more 30 fetal tissues (those displaying the larger weight reduction) for the control group were hybridized to long oligonucleotides microarrays, at the Nimblegen platform in Reykjavik, Iceland, following described protocols. A total of 390,000 oligonucleotides representing 23,456 transcripts from the rat genome were hybridized with fluorescently labeled cDNAs from each fetal tissue (LP group vs control group). Image analysis was performed with the NimbleScan software (Nimblegen), and feature intensities were exported as .pair files. ArrayStar 3.0 (DNASTAR, Madison, WI) was used for probe summarization and normalization (RMA algorithm, quantile normalization), statistical analysis of differentially expressed genes (Student's t-test with Benjamini-Hochberg false discovery rate correction) and gene ontology analysis. The entire microarray data set is available at the Gene Expression Omnibus (accession n° GSE25764).

### Quantitative Real-Time RT-PCR

Total RNA was treated with DNAse. DNAse was inactivated and cDNA synthesis was performed using the MMLV cDNA synthesis kit (Invitrogen, Carlsbad, CA). Mix preparation and PCR amplification were performed according to standardized protocols using Platinum® Quantitative PCR MasterMix (Invitrogen, Carlsbad, CA) and Light Cycler (Roche, Indianapolis, IN) respectively. PCR oligonucleotides were chosen using PRIMER3 (http://frodo.wi.mit.edu/cgi-bin/primer3), and designed from the coding sequence of each gene of interest and generally overlapping two consecutive exons. The different couples were aligned against the rat genome using BLAST to check their specificity. 18S mRNA amplified from the same samples served as an internal control. Quantitative PCR was carried out from 2 µl of a 10 µl dilution of the cDNA in a volume of 17 µl using a LightCycler thermocycler (Roche, Indianapolis, IN). The master mix SYBR Green from Invitrogen was complemented with MgCl2 (4 mM final concentration), BSA (0.05 g/l) and primers 10 µM). The PCR program was as follows: 50C 120s, 95C for 120 s, 35 cycles of 94°C for 5 s, 58°C for 10s and 72°C for 30 s, followed by 65°C for 15 s and a raise in temperature to 99°C at 0.1°C /s. This last step was used to generate fusion curves. The specificity of the PCR products was verified by melting curve analyses and agarose gel electrophoresis, as previously described [23]. Ct values were calculated with LightCycler Software and The Ct values of the gene of interest were normalized by the Ct value obtained for the 18S gene taken as a reporter gene, using the 2-ΔΔCt approach as outlined by Livak and Schmittgen [24].

### Microarray analysis

Non-supervised clustering analysis was performed using Cluster [25] and JavaTreeview [26]. This analysis clusterized reliably the duplicates of each hybridization and also the various organs either from growth-restricted or normal newborns (Figure S6), marking the good technical quality of the data obtained and analyzed throughout this study. Pathway analysis was carried out using the GePS tool of the Genomatix portal (http://www.genomatix.de/en/index.html). Table of genes modified more than twice were submitted as text files with the level of induction/repression. This made it possible to generate pathways with a threshold for significance in the gene clustering established at p<0.01.

### Promoter analysis

Promoters were recovered using the Genomatix ™ software and database. Predicted transcription factor binding sites were identified and counted. Statistical analysis was performed by the comparison of induced and repressed genes using a Student t-test corrected for multiple testing.

### Statistical analysis for abnormal representation of groups of genes

When groups of genes (epigenetic regulator, imprinted genes, per chromosome genes) were analyzed they were compared to the rest of the genome using a χ2 contingency test. Difference were considered significant when p<0.05.

## Results

### 1. Organ-specific intensity of the effects of growth restriction

**Figure 1:**
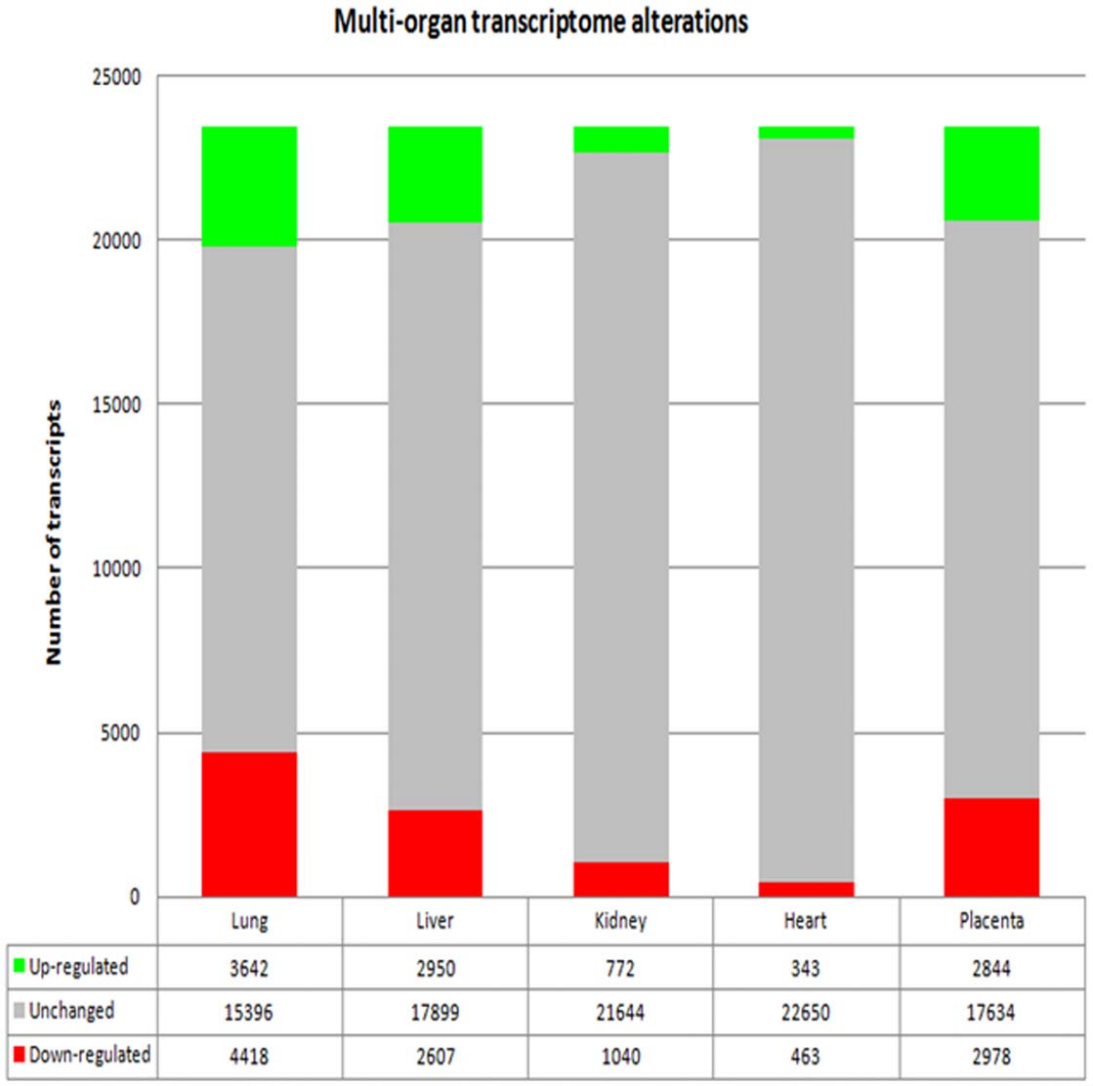
Alterations of the transcriptome in the five organs analyzed in this study. mRNA transcripts that are increased in Intra-Uterine Growth Restriction are represented in green, decreased transcripts are in red. The chosen threshold is 2-fold, as described in the text. There appears to be a biased excess in decreased transcripts in lungs, kidney and heart, a biased excess of increased transcripts in the liver, no significant difference in the placenta (see text).

An estimation of the intensity of the transcriptional alterations was made using a similar threshold fixed at two folds throughout the complete study, starting from the 23456 transcripts that were present in the microarray (Figure 1). The microarray data were validated by qRT-PCR for a series of 12 genes in lungs and liver and 11 genes in heart, as shown in Figure S1; the expression value for the q-RT PCR are the average of four independent experiments carried out on pools of cDNA. The validation for placenta and kidney was previously published [21]. The correlation between the microarray and the qRT-PCR was >0.95, while the determination coefficient was in all cases>0.8.

We could classify the organs relatively to the percentage of modified genes. In the lungs, it reached ∼34%, ∼24% in the placenta and liver, ∼7.7% in kidneys and only 3.3% in the heart.

Only the placental gene expressions were not deviated towards up- or down regulation (p = 0.39), while it appeared that lung, kidney and heart presented an increased number of down-regulated genes (p<2.7 10^−17^, <1.5 10^−10^, <1.2 10^−4^, respectively). By contrast, the liver presented an excess of up-regulated transcripts (<2.1 10^−5^).

### 2. Clustering of the effects reveals a dichotomy in organ response to stress

**Figure 2:**
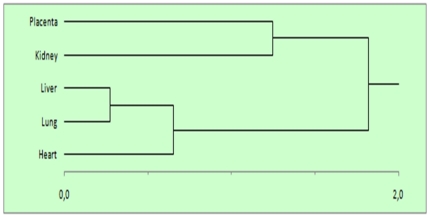
Non-supervised clustering of correlations between tissues categorized placenta and kidney into one group, while liver, lung and heart are into another group. This is due to the fact that the transcriptional response to IUGR is often opposite between the two groups of organs. This is illustrated in more details in Table 1.

Non-supervised hierarchical clustering was performed from the correlation coefficients calculated between the different organs and two clusters were separated, one including placenta and kidney, and the other including heart, lung and liver (Figure 2). The cophenetic correlation, which grossly measures the robustness of the tree was of 0.993 (p value = 1.3 10^−8^). This inverse correlation is illustrated in Table 1, for instance between placenta and lung. Statistically the number of genes that should be over-expressed more than twofold in both these tissues was estimated at 442 (grey part under the diagonal), while the actual number of genes in this situation was only 26. Reciprocally, the number of genes that should be reduced more than twofold in both tissues is estimated at 561, while the actual number is only 125. By contrast, the number of genes that should be induced more than two-fold in the placenta and reduced more than two-fold in the lung can be estimated at 536, while the actual number of genes in this situation is actually 1556; finally, in the reciprocal case (induced more than two-fold in lungs and reduced to the same extent in the placenta), the estimated number of genes is 462 while the actual value is 820. Thus, but some rare exceptions, the use of thresholds yielded the same type of results than the global correlation analysis, and confirmed the grouping in two categories of expressional response to induced IUGR, one of the kidney/placenta type and one of the liver/lung/heart type.

**Table 1:** Concordances and discordances between up and down reulated genes in the five tissues analyzed.

		Up-regulated					Down-regulated				
		Lung	Liver	Kidney	Heart	Placenta	Lung	Liver	Kidney	Heart	Placenta
Up-regulated	Lung	3642	**1987**	*20*	**256**	*26*	0	*43*	**688**	*16*	**1556**
	Liver	_459_	2956	*12*	**246**	*151*	_557_	0	**614**	*43*	**1091**
	Kidney	_120_	_97_	772	2	**164**	_145_	_86_	0	**22**	*28*
	Heart	_53_	_43_	_11_	343	*1*	_65_	_38_	_15_	0	133
	Placenta	_442_	_358_	_94_	_42_	2844	_536_	_316_	_126_	_56_	0
Down-regulated	Lung	0	*77*	**261**	*6*	**820**	4418	**1219**	*24*	**170**	*125*
	Liver	_405_	0	**258**	*6*	*353*	_491_	2607	*14*	**109**	*163*
	Kidney	_161_	_131_	0	**123**	*0*	_196_	_116_	1040	*8*	**733**
	Heart	_72_	_58_	_15_	0	*38*	_87_	_51_	_21_	463	*42*
	Placenta	_462_	_375_	_98_	_44_	0	_561_	_331_	_132_	_59_	2978

The small numbers, below the diagonals corresponds to the expected number of genes, calculated according to a contingency table, bold or italics mark an excess or deficit in the number of genes actually counted in each category, respectively.

### 3. Analysis of specific groups of genes

We then used our dataset to investigate the impact of the applied nutritional stress on genes susceptible to carry at least partly a ‘memory’ of the modifications, namely epigenetic regulator genes, taken in a broad sense and imprinted genes. We also performed a chromosome-based analysis in order to detect possible biases in deregulation according to the chromosome analyzed, susceptible to occur according to previous results [27].

#### a. Epigenetic regulators

We investigated five groups of genes, encoding DNA methyltransferases, Histone Deacetylases, Histone methyl- and acetyl-transferase, Bromodomain-containing proteins recognizing acetylated lysine residues in particular in histone tails [28], and Chromodomain-containing proteins recognizes methylated histones [29] and appears in the RISC complex [30]. These proteins are important epigenetic regulators associated either with opened chromatin or closed chromatin for bromodomains and chromodomains, respectively. The complete list of genes analyzed is given as Table S1.

**Figure 3:**
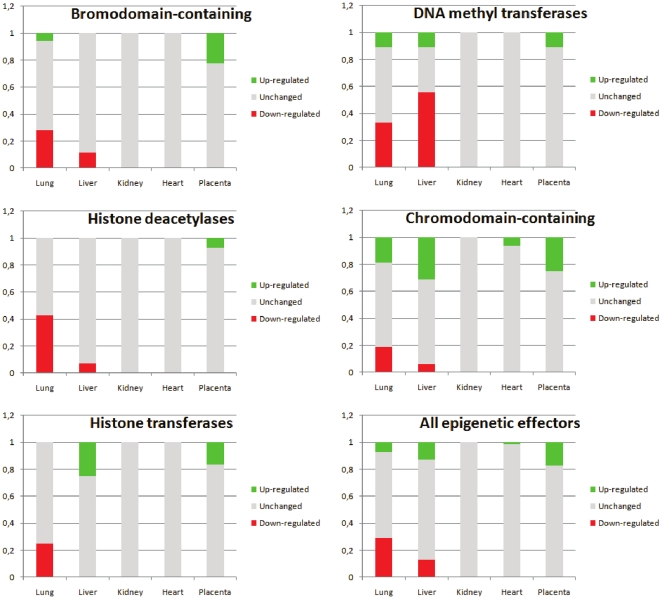
Genes modulating epigenetic modifications of the chromatin are differentially altered according to the tissue under scrutiny. The list of transcripts analyzed is given as Table S1. Note that in the placenta, all these effectors are up-regulated, while the trends tends to be opposite in the lungs. These alterations are not strictly correlated with an ‘opened’ or a ‘closed’ chromatin; for instance, the up-regulation of histone transferases genes in the liver concerns acetyl- and methyl-transferases, altogether.

The results are summarized in Table 2 and Figure 3. DNMTs were overall down-regulated exclusively in the liver (p = 0.00011) while HDACs are generally down-regulated genes in the lungs. No significant values were seen for genes encoding bromodomain- or chromodomain- containing proteins, or for histone either acetyl- or methyl- transferases. However, when all the categories were taken together, the statistical tests were significant for the lung and for the placenta. In this latter organ, the expression of genes encoding epigenetic modulators could never be found reduced, meaning that seemingly, the epigenetic machinery is fully activated in this organ, emphasizing its major role as an interface between the stressful environment and the foetus.

**Table 2:** Number of genes involved in epigenetic regulation by tissue and by category in IUGR rats.

All genes	Lung	Liver	Kidney	Heart	Placenta	χ2 Lung (p value)	χ2 Liver (p value)	χ2 Kidney (p value)	χ2 Heart (p value)	χ2 Placenta (p value)
Up-regulated	3642	2950	772	343	2844					
Down-regulated	4418	2607	1040	463	2978					
Unchanged	15396	17899	21644	22650	17634					
**DNMTs**	Lung	Liver	Kidney	Heart	Placenta	0.5337	**0.0001**	0.6861	0.0949	0.5028
Up-regulated	1	1	0	0	1					
Down-regulated	3	5	0	0	0					
Unchanged	5	3	9	9	8					
**HDACs**	Lung	Liver	Kidney	Heart	Placenta	**0.0367**	0.2921	0.5565	0.8520	0.2664
Up-regulated	0	0	0	0	1					
Down-regulated	6	1	0	0	0					
Unchanged	8	13	14	14	13					
**CHDs**	Lung	Liver	Kidney	Heart	Placenta	0.9366	0.0754	0.5119	0.7795	0.1215
Up-regulated	3	5	0	1	4					
Down-regulated	3	1	0	0	0					
Unchanged	10	10	16	15	12					
**BRDs**	Lung	Liver	Kidney	Heart	Placenta	0.3832	0.2676	0.4708	0.2430	0.1486
Up-regulated	1	0	0	0	4					
Down-regulated	5	2	0	0	0					
Unchanged	12	16	18	18	14					
**Histone Methyl/acetyl transferases**	Lung	Liver	Kidney	Heart	Placenta	0.3222	0.2457	0.6051	0.7260	0.3998
Up-regulated	0	3	0	0	2					
Down-regulated	3	0	0	0	0					
Unchanged	9	9	12	12	10					
**All epigenetic effectors**	Lung	Liver	Kidney	Heart	Placenta	**0.0328**	0.8633	0.0557	0.4990	**0.0044**
Up-regulated	5	9	0	1	12					
Down-regulated	20	9	0	0	0					
Unchanged	44	51	69	68	57					

Significant p values, calculated by a χ2 contingency test are indicated in bold characters. The numbers represent the count of modified and not modified genes for each category and each tissue analyzed (see also figure 1).

Interestingly, Dnmt3a and 3b are strongly downregulated in the lungs (−11.3 and −3.14 fold respectively), suggesting an overall unability of this organ to *de novo* methylate CpG islands. In addition, in lungs, genes involved in the monocarbon and glutathione metabolism pathways were almost systematically down-regulated. In particular we found that Betaine homocystein methyltransferase (BHMT), cystathionase (CTH), cysteine sulfinic acid decarboxylase (CSAD), methionin synthase (MTR), cystathionine beta synthase (CBS), Serine hydroxymethyltransferase1 (SHMT1), dihydrofolate reductase (DHFR) were reduced −3, −1.8, −3 −1.9, −2.4, −3.3, −1.9 and −2 fold respectively. This suggests that the availability of methyl residues may be limited in the lungs. Together with the overall downregulation of DNMTs, this indicates that de novo DNA methylation is probably quite limited in the lungs. This does not eliminate the possibility of a passive demethylation affecting all the genome in this organ, with possible long-term consequences. We observed the inverse tendency for these genes in the placenta (data not shown). It is interesting to notice that alterations are not directly linked to the generation of an ‘opened’ or ‘closed’ state of the chromatin.

#### b. Imprinted genes

Several imprinted genes have been shown to be altered at the expression level following embryo manipulation and placental diseases [27], [31] prompting us to analyze them specifically. Known imprinted genes were recovered from the Otago catalogue [32], at http://igc.otago.ac.nz/home.html. Since the analyses are based upon a rodent model, the mouse list was used to screen the Nimblegen™ rat array. A total of 83 transcripts was obtained (Table S2).

**Figure 4:**
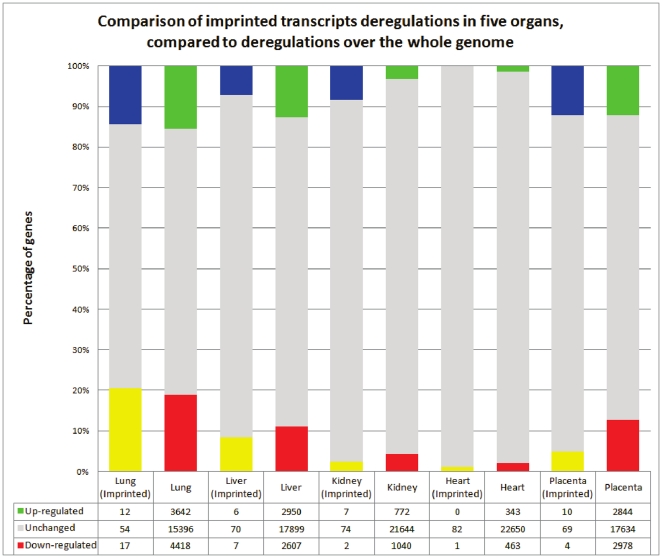
Comparison of the deregulations of known imprinted genes with the genomic alterations, 83 transcripts obtained from the Otago databasehttp://igc.otago.ac.nz/home.html. The color code is the same than the other figures for the pan-genomic alterations, blue and yellow were added for imprinted genes when they are induced or repressed, respectively. The existence of significant discrepancies between imprinted genes and the rest of the genome especially in the kidney and to a lesser extent in the placenta is discussed in the text.

The results of the comparison between imprinted genes and the rest of the genome is presented in Figure 4. Statistical analyses by χ2 tests showed a significant deviation only for the kidney, where up-regulated genes were in excess compared to the rest of the genome, while down-regulated were on the contrary in lower than expected number (p = 0.024). In this organ, the down-regulated genes were *Dlk1* and *Dmd* (3 fold and 2 fold, respectively), while the up regulated genes were *Mest*, *Gnas*, *Ndn*, *Sfmbt2*, *Apoe*, *Th* and *Nnat*, induced 2.1, 2.6, 2.8, 3.5, 3.9, 3.9 and 4.6 fold, respectively. In the placenta, if the genes are divided into two classes (reduced more than twice and the rest), the p value is 0.031, suggesting that overall, there is a deficit in down-regulated imprinted genes in the IUGR placenta at term. This is reminiscent of the effects of In vitro Fertilization techniques applied to mouse blastocyst, which triggers a selective activation of the expression of imprinted genes [27].

#### c. Chromosome-specific alterations of gene expression

**Figure 5:**
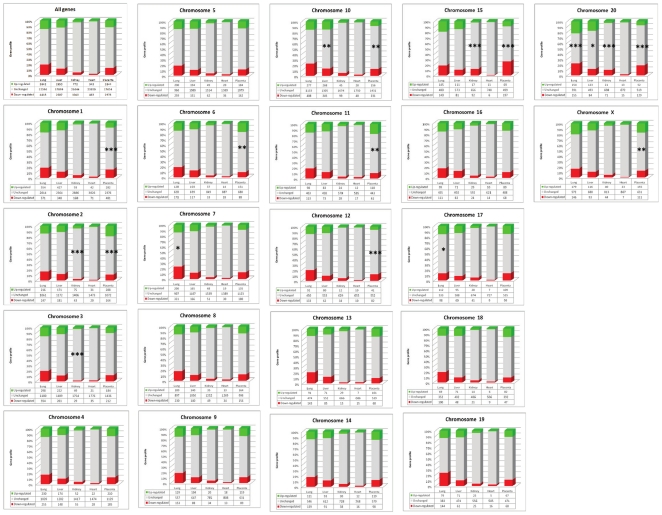
Transcriptome alterations chromosome per chromosome and organ per organ. Significant discrepancies in the number of modified genes were observed, especially for 12 chromosomes, and are much more abundant in the placenta than in other organs (9 chromosomes affected amongst 21). It is interesting to notice that while there is as many deregulations in the liver as in the placenta, no such chromosomal grouping could be observed, since in this organ, only chromosomes 10 and 20 contains clusters of deregulated genes, detected with a moderate level of significance (** and *, respectively). In the heart, no chromosome-specific cluster of deregulated genes could be observed. Asterisks represent significance thresholds (*: <0.05, **: <0.01, and ***: <0.001). Tests were performed using a c2 contingency test followed by a Bonferronni correction for multiple testing (actual values are listed in Table 3).

**Figure 6:**
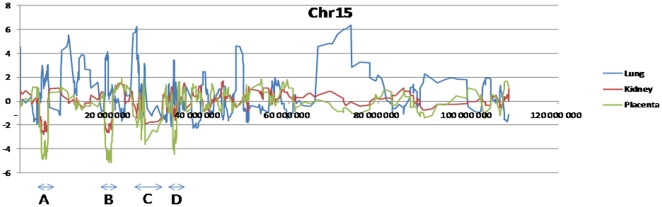
Profile of gene expression deregulation for three organs on chromosome 15. We coud identify four windows where gene expression dergulations were opposed between lungs (blue line) on the one hand and kidney-placenta in the other hand (red and green line, respectively). Deregulations are represented as the average of log_2_(gene induction ratios), on windows of ten consecutive genes. Four regions named A, B, C and D are analyzed in the text in terms of gene content.

We analyzed the effects of low protein maternal diet induced-IUGR in the rat model on a chromosome per chromosome basis, to identify possible bias in abnormal gene expression (Table 3 and Figure 5). After Bonferronni correction for multiple testing there appears a bias in 18 cases, half of these cases concern the placenta. The strongest biases were found for chromosome 15, for the Placenta and the Kidney. In these organs, the explanation for the discrepancy resides in the concentration of clusters of deregulated genes (Figure 6). As shown in the figure, which is drawn after a smoothing using a sliding window of 10 consecutive genes, the discrepancy with the rest of the genome is mainly due to the existence of four consecutive regions (A, B, C and D on Figure 6). The down-regulated genes behave in a strikingly similar way between the kidney and placenta, suggesting that the same type of expressional regulation might be at work in placental and renal tissue, as suggested by the hierarchical classification. The first cluster (A) of down-regulated genes is located between 4.6 and 6.3 megabases on chromosome 15. It encompasses genes of the Spetex family (essentially known to be involved in spermatogenesis) that probably share regulatory sequences in their promoters. The second region (B) is located between 19.3 and 20.6 megabases and encompasses a second cluster of genes of the Spetex family. The third cluster maps between 27.0 and 35.3 megabases, and can be in fact subdivided into a series of three gene clusters; the first (27–27.2) is composed of a series of Rnases and of the two angiogenins (Ang1 and Ang2), that are all under-expressed in the placenta; the second (27.9–30.8) encompasses genes of the T-cell receptor family; the third (34.1–35.3) encompasses a cluster of genes of the mast cell protease family, immediately followed by a series of granzyme-encoding genes. Overall, the level of deregulation is much higher in the placenta. To illustrate the existence of a “mirror” regulation of lung and liver compared to kidney and placenta, lung deregulations were represented on the figure for the lung, even though these are not different from the rest of the genome for this specific organ. While some clusters are really in mirror, such as clusters A and B, it clearly appears that lung-specific deregulations exist, such as a long series of up-regulated genes spanning from 75 to 80 Megabases on Chr 15. In this specific case, however, the region is spanning the centromere and contains very few genes (only 28 on 20 Mb), the apparent up-regulation being the result of a limited number of very strongly up-regulated genes in the region, that gives this impression.

**Table 3:** Distribution biases of deregulated gene broken down per chromosome after Bonferronni correction.

	*Lung*	*Liver*	*Kidney*	*Heart*	*Placenta*
Chr1	0,93719715	27,7616851	16,9094284	50,9431964	**5,8001E-07**
Chr2	0,56963298	24,4103093	**0,00528531**	4,35429977	**5,7061E-12**
Chr3	57,592184	86,0966746	**1,0359E-06**	56,5107586	0,65191661
Chr4	8,79755304	7,33832422	32,2382249	97,7573529	15,8225724
Chr5	21,2714321	49,1904907	99,3886778	36,5643412	28,4298196
Chr6	39,9012864	19,1931742	25,0329042	103,759756	**0,00398762**
Chr7	**0,03372224**	46,6923504	29,3933753	91,6593564	0,69234961
Chr8	18,0181441	8,79806359	21,1989756	12,4593178	68,3992631
Chr9	93,3072846	86,9995348	30,7128232	20,2238963	6,3430038
Chr10	0,07739599	0,00747273	2,70908847	80,9597416	**0,00345094**
Chr11	98,310212	64,5104577	28,7471647	23,6581667	**0,00337356**
Chr12	40,7190874	22,4247406	7,71631187	69,3864974	**0,00063281**
Chr13	13,9540592	12,1852239	0,23267013	59,2385059	2,04433468
Chr14	22,9582733	78,0615511	70,8415389	104,143738	5,98657976
Chr15	3,82645274	29,0830732	**7,4459E-20**	6,61142125	**4,8548E-23**
Chr16	57,4235089	27,6180086	18,4202361	96,8714551	15,9626296
Chr17	**0,02793192**	3,4775979	28,8720671	17,2061379	8,56081387
Chr18	37,8838928	35,6738566	70,0097025	81,2997108	0,44847596
Chr19	0,55972949	57,1658765	52,5183901	23,7079137	37,0946765
Chr20	**1,1811E-05**	**0,02251991**	**1,1301E-09**	69,0101162	**3,2653E-05**
ChrX	0,08333262	80,2258047	12,7409338	0,13210437	**0,00236127**

Significant p values, calculated by a χ2 contingency test are indicated in bold at the threshold of 0.05 after Bonferronni correction for multiple testing. The numbers represent the p value times the number of tests performed, which explains why some values are above 1.

### 4. Pathway analysis using GePS (Genomatix™)

Using Genomatix GePS, we searched for pathways significantly enriched in modified genes in the different organs under scrutiny. The exhaustive discussion of these pathways is given as Material S1. The most striking observations are the identification of the VEGFR1 pathway in the placenta (Figures S2A and S2B; note, colors in Figures S2, S3, S4 and S5 correspond to up-regulated genes in red and according to the intensity, and blue for down-regulated genes), of coagulation cascades in the kidney, confirming with novel analysis tools the pathways previously observed [21] (Table 4A), of FOXA transcription factor regulation in the heart, BAD signaling in the lungs (Table 4 B) and PKA signaling in the liver.

**Table 4:** Genes modified and clustered by Genomatix in pathways in the kidney and in the lung, with highly significant p-values.

Kidney Canonical pathways	Pathway id	P-value	Adjusted p-value	# Genes (observed)	# Genes (expected)	# Genes (total)	List of observed genes
intrinsic prothrombin activation pathway	BioCarta:intrinsic pathway	4,62E-13	n/a	12	0.712	24	KNG1, FGG, F12, F11, PROC, F13B, F10, FGB, F2, F9, FGA, KLKB1
extrinsic prothrombin activation pathway	BioCarta:extrinsicpathway	1,16E-09	n/a	8	0.415	14	FGG, PROC, F13B, F10, FGB, F2, TFPI, FGA
fibrinolysis pathway	BioCarta:fibrinolysis pathway	1,49E-07	n/a	7	0.475	16	FGG, PLG, F13B, FGB, F2, FGA, CPB2
classical complement pathway	BioCarta:classic pathway	1,01E-03	n/a	4	0.475	16	C5, C8A, C1QC, C9
alternative complement pathway	BioCarta:alternative pathway	2,61E-03	n/a	3	0.297	10	C5, C8A, C9
platelet amyloid precursor protein pathway	BioCarta:plateletapp pathway	5,84E-03	n/a	3	0.386	13	PLG, F2, F9
lectin induced complement pathway	BioCarta:lectin pathway	7,27E-03	n/a	3	0.416	14	C5, C8A, C9
mechanism of gene regulation by peroxisome proliferators via ppara	BioCarta:ppara pathway	7,39E-03	n/a	5	1.247	42	EHHADH, FABP1, APOA2, LPL, PPARA

Lung Canonical pathways	Pathway id	P-value	Adjusted p-value	# Genes (observed)	# Genes (expected)	# Genes (total)	List of observed genes
AKT(PKB)-Bad signaling	INOH:EPO_signaling	1,49E-05	n/a	61	37.28	178	RPS6KA2, EPO, ACVR1B, BRAF, IRAK4, MAP3K10, PDGFRB, PRKCI, STAT3, RPS6KB2, GFRA1, MAPK8, PRKCZ, ACVRL1, MAP2K1, PIK3CB, PRKCE, PRKCQ, JAK1, KIAA1804, EGFR, MET, IKBKE, BTK, MAPK12, MAP2K7, STAT5A, NTRK1, MAP4K2, RIPK3, SYK, MAPK13, PTK2B, TEC, CSNK1G3, INSR, PRKCG, FGFR3, ADRBK1, PRKCD, PIK3CD, CDK6, PLK3, MAP3K11, FLT4, MAP3K12, GRK4, FGFR1, CAMK2B, PIK3R2, FGFR4, ARAF, ACVR2B, EPOR, MAP3K5, CCNH, FYN, SRC, MAP3K14, MAP3K2, CSNK1G2
IL-7 signaling pathway(JAK1 JAK3 STAT5)	INOH:IL-7_signaling	5,29E-05	n/a	59	37.07	177	RPS6KA2, ACVR1B, BRAF, IRAK4, MAP3K10, PDGFRB, PRKCI, RPS6KB2, GFRA1, MAPK8, PRKCZ, ACVRL1, MAP2K1, PIK3CB, PRKCE, PRKCQ, JAK1, KIAA1804, EGFR, MET, IKBKE, BTK, MAPK12, MAP2K7, STAT5A, NTRK1, MAP4K2, RIPK3, SYK, MAPK13, PTK2B, TEC, CSNK1G3, INSR, PRKCG, FGFR3, ADRBK1, PRKCD, PIK3CD, CDK6, PLK3, MAP3K11, FLT4, MAP3K12, GRK4, FGFR1, IL7R, CAMK2B, PIK3R2, FGFR4, ARAF, ACVR2B, MAP3K5, CCNH, FYN, SRC, MAP3K14, MAP3K2, CSNK1G2
Migration	INOH:VEGF	9,14E-05	n/a	59	37.70	180	ELK1, RPS6KA2, ACVR1B, BRAF, IRAK4, MAP3K10, PDGFRB, PRKCI, RPS6KB2, GFRA1, MAPK8, PRKCZ, ACVRL1, MAP2K1, PIK3CB, PRKCE, PRKCQ, JAK1, KIAA1804, EGFR, MET, IKBKE, BTK, MAPK12, MAP2K7, NTRK1, MAP4K2, RIPK3, SYK, MAPK13, ITPR3, PTK2B, TEC, CSNK1G3, INSR, PRKCG, FGFR3, ADRBK1, PRKCD, PIK3CD, CDK6, PLK3, MAP3K11, FLT4, MAP3K12, GRK4, FGFR1, CAMK2B, FGFR4, ARAF, PLCG2, ACVR2B, MAP3K5, CCNH, FYN, SRC, MAP3K14, MAP3K2, CSNK1G2
Heterotrimeric GPCR signaling pathway (through glutamate, G alpha q and PLC beta)	INOH:GPCR_GroupI_metabotropic_glutamate_receptor	1,35E-04	n/a	15	5.86	28	GRM5, BRAF, PRKCI, PRKCZ, PLCB2, MAP2K1, PRKCE, PLCB1, PRKCQ, PLCB3, ITPR3, PRKCG, PRKCD, GNB3, ARAF
Cytokine receptor degradation signaling	INOH:JAK_STAT_pathway_and_regulation	5,77E-04	n/a	79	56.97	272	IL13, RPS6KA2, EPO, IL12A, ACVR1B, BRAF, IRAK4, MAP3K10, IL1RAP, PDGFRB, PRKCI, PTPN6, STAT3, RPS6KB2, IFNA7, GFRA1, MAPK8, PRKCZ, IL6R, ACVRL1, MAP2K1, TNF, PRKCE, CSF3R, PRKCQ, SOCS7, JAK1, KIAA1804, EGFR, MET, PRLR, IKBKE, IL12RB2, BTK, TNFSF10, MAPK12, CSF3, MAP2K7, STAT5A, IL6ST, NTRK1, IL12RB1, MAP4K2, RIPK3, SYK, MAPK13, PTK2B, TEC, CSNK1G3, INSR, PRKCG, FGFR3, ADRBK1, PRKCD, CDK6, PLK3, MAP3K11, FLT4, MAP3K12, GRK4, FGFR1, IL7R, CAMK2B, FGFR4, ARAF, IL9, ACVR2B, LTA, EPOR, MAP3K5, CCNH, FYN, SRC, MAP3K14, MAP3K2, MPL, CSNK1G2, IFNAR1, GRB2
Thromboxane A2 receptor signaling	NCI-nature:txa2pathway	1,38E-03	n/a	21	11,100894	53	EGF, PRKCZ, ICAM1, PLCB2, PRKCE, PRKCQ, BLK, GNA12, NOS3, EGFR, GNA11, ARHGEF1, SELE, SYK, PRKCG, ADRBK1, PRKCD, HCK, FYN, SRC, FGR
amb2 Integrin signaling	NCI-nature:amb2_neutrophils_pathway	2,71E-03	n/a	20	10,89144317	52	MMP2, SELPLG, PRKCZ, ICAM1, JAM3, TNF, THY1, CTGF, PLAU, BLK, MST1R, PLG, APOB, LRP1, ITGB2, MMP9, HCK, FYN, SRC, FGR
basic mechanism of action of ppara pparb(d) and pparg and effects on gene expression	BioCarta:pparpathway	7,56E-03	n/a	4	1,047254151	5	PTGS1, PPARG, PPARA, PPARD
HIV-1 Nef: Negative effector of Fas and TNF-alpha	NCI-nature:hivnefpathway	7,70E-03	n/a	14	7,330779055	35	RELA, BIRC3, MAPK8, CASP2, TNF, CASP9, FAS, MAP2K7, DFFB, CD247, BID, CFLAR, MAP3K5, MAP3K14

Significant p values for the most significant cluster of genes were given by GePS (Genomatix). The p value is calculated by comparing the actual number of modified genes observed in the arrays with the expected count from the total number of genes present in a given biological function. Significant values reflect a strong enrichment.

**Figure 7:**
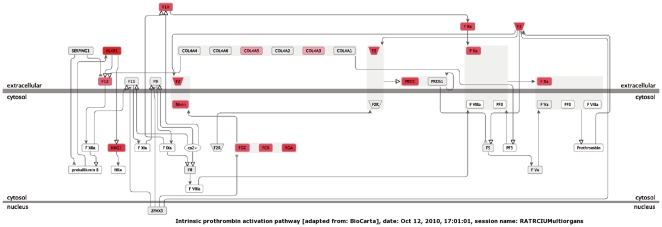
An example of pathway significantly enriched in genes modified by intra-uterine Growth-restriction in the kidney, the intrinsic pro-thrombin activation pathways. The list of genes with their induction levels were submitted to GePS (Genomatix™). Genes that are up-regulated are in red, according to the level of modification, no down-regulated genes could be identified in this pathway. Note the systematic induction of fibrinogen peptids (fibrinogen A, B and C). The overall effects of the activation of this cascade should be to induce renal clotting and coagulation, and thus to perturb kidney function and organogenesis, especially if there is a lesion.

The activation of VEGF-associated cascades in the IUGR placenta, suggests an attempt toward at least a compensation of the shortage in nutrients by an improved vascularisation of the placenta. In the IUGR kidney, the intrinsic prothrombin pathway is shown (Figure 7); numerous coagulation factors are consistently up-regulated, demonstrating a severe alteration of this pro-coagulation cascade, and suggesting an increased risk of thrombosis and clotting. The induction levels ranged from 2.2 for COL genes to ∼6–7 fold for Kallikrein 1 and FGA.

### 5. Promoter analysis

**Figure 8:**
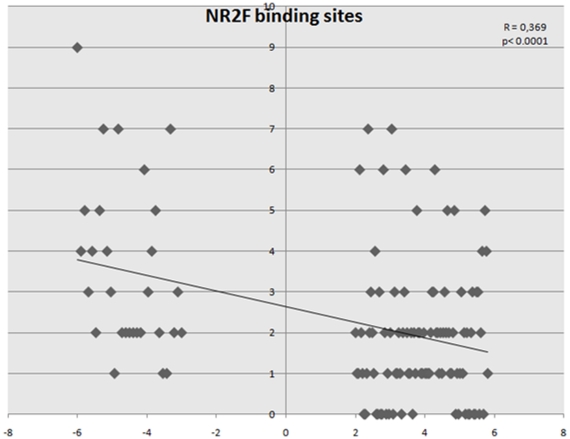
Promoter composition analysis in the various organs suggests an increased occurrence of putative binding sites for the transcription factor NR2F in promoters of genes down-regulated in IUGR. Promoter regions were collected (all the alternative promoters with defined Transcription Start Sites) using the Genomatix option Gene2Promoter for the 20 most induced and 20 most repressed genes. Each dot corresponds to a promoter. In ordinates are the numbers of putative binding sites for NR2F, and in abcissae the log_2_ of the induction ratio for each promoter analyzed. Since one gene has generally several promoters, a unique induction ratio was considered for these promoters for the sake of simplicity.

In an attempt to identify master genes of the IUGR-induced deregulations, we proceed to an analysis of the predicted binding sites for transcription factors present in genes oppositely altered. The occurrence of binding sites for transcription factors was obtained from the Matinspector Genomatix software for the promoters of the 20 most induced and 20 most repressed genes. Then a Student T-test analysis was carried out to compare the occurrences, after Bonferronni correction for multiple testing as already described for placenta and kidney [10], [21]. Significant differences in the occurrence of putative transcription factor binding sites were found only in the liver for NR2F, KLFS and PAX5 (Figure 8). We checked the transcriptome data for NR2F binding factors expression (HNF4A, HNF4G, NR2C, NR2F) and for KLFS binding factors (KLF proteins), and could show that KLF4, 5, 7 are expressed at an intermediate level in the liver and are down-regulated 2 to 3 fold by IUGR, suggesting that a decrease level of specific KLFs could participate in the reduced expression level of down-regulated genes. HNF4A and HNF4G are induced 1.83 and 1.84. It could be that these transcription factors play a negative regulatory action on the promoters of the down-regulated genes. This could be consistent with recent data showing that repressive function of HNF4 proteins is achieved with specific co-repressors [33].

## Discussion

Intra-Uterine Growth Restriction is a major health problem in humans, combining risks for the baby and contributing to an increased susceptibility to adult systemic diseases by the birth of low birth weight newborns [34]. IUGR is multifactorial, but animal models reproducing several aspects of the disease, have been developed to analyze the underlying biological and cellular defects [35]. Taking advantage of high-throughput technologies is an efficient way of getting a full vision of the consequences of this disease [21], but today these approaches focused specifically on one organ [36]. To the best of our knowledge, our study is the first that addresses abnormal expression profiles in IUGR simultaneously in different tissues of embryonic and extra-embryonic origin and documents thoroughly these alterations, in a diet-induced model of fetal growth restriction. Additional studies on other tissues that can be important for programming later consequences on health, would be of great interest but were not treated in the present study. In the future in particular, analyzing pancreas islets would be an interesting perspective, since reduction in their size caused by IUGR have been associated with the development of type 2 diabetes [37], [38]. In addition, it has been shown recently that these anomalies are associated with specific epigenetic signatures involving abnormal methylation of specific promoter sequences [39]. It could be also interesting to extend our study to fat tissue, and in this case to distinguish subcutaneous fat from perivascular fat that behaves differently in terms of gene expression [40]. Nevertheless our study allows drawing specific conclusions.

The major conclusions of our work are that organs do not react similarly to protein depletion that induces Intra-Uterine Growth Restriction. This is true both concerning the intensity of the deregulations that varies on a range of 10 fold (from 3.3% of genes modified at the threshold of 2 in the heart to 34% in the lung), and for the profile of the responses that places placenta and kidney in one group, while liver, lungs and heart are in another group showing that two organs that respond similarly may have large variations in the intensity of the response, while two organs that respond oppositely such as liver and placenta, may have a similar number of modified genes. Our data may thus suggest that the long-term effects and severity of protein restriction could vary according to the organs, some being more sensitive targets. Interestingly also, long-term consequences of low birth weight are not clearly described for the heart (while they are demonstrated for the vascular system); the long-term effects on the kidney may rather be due to a deficiency in glomerular number than specifically to transcriptional alterations. The strong effect on the liver is consistent with the metabolic syndrome, while lung dysfunction in adults is not thoroughly documented for small birth weight babies [41], although a mild effect has been described in one study [42] . There does not appear to be a link between the embryonic origin of the tissues and the sense (or intensity) of the transcriptional alterations; for instance kidney and heart that are both of mesodermal origins are clustered in different groups. Coming back to the embryology of organ function, we searched for correlations between developmental chronology and IUGR-induced expression alterations. Liver developmental timing was studied by Godlewski and coworkers using the Carnegie stages reference. The study showed that liver becomes functional from stage 18-23 [43]. Heart starts to function 21 days post-conception in humans, corresponding to Carnegie stages 9–10, kidney structures are considered to start forming around 10–12 days of gestation in mice (26 days in humans) but will be completed only 14–17 days post-birth. Renal function nevertheless starts from the 4th week of gestation (Carnegie stage 10–11). When kidneys are formed, the liver is only present as a bud. Lungs start obviously to function postnatally and alveolisation goes on up to the third postnatal week in rats. In sum, we can conclude that for embryonic structures, the intensity of the transcriptional alterations is apparently strongly associated with the developmental timing of each studied organ, and closely depends on its function, rather than on its development. When an organ is set up at an early stage, such as the heart, its transcriptional suppleness is drastically limited, while when its function is delayed, such as the lungs, intense transcriptional modifications can occur. In our study, we also address the important question of the permanence of these alterations in adult life which could be stabilized by epigenetic modifications, known to occur for instance in the cord blood [44], or the placenta [23], [45], [46]. It is important to notice that the placenta as a transitory structure may accumulate adaptive epigenetic modifications that will not have any consequence on adult life. For permanent organs, such as the liver it may be possible that epigenetic alterations apply, but on a strictly limited range of gene targets; overall, in this organ it appears that DNA methyltransferases are reduced, and our results comforts the data of Lillycrop and coworkers on this organ showing a reduced expression of hepatic DNMT1 in rats originating from a low protein diet given to their mothers [47]. Feeding a low-protein diet to pregnant rats causes hypertension and endothelial dysfunction in the offspring [48], [49]. These alterations are accompanied by metabolic and gene expression changes, including overexpression of the hepatic glucocorticoid and PParα receptors, and epigenetic changes that facilitate transcription of these receptors: For the glucocorticoid receptor, these changes include histone modifications as shown in a model of IUGR obtained by bilateral uterine ligation [50]. IUGR has also been shown to down-regulate DNMT1 expression in the rat brain [51]. Epigenetic control of gene expression mediated by cytosine methylation in DNA, histone modifications, and chromatin remodelling plays a critical role in proliferation, cell cycle withdrawal, and terminal differentiation [52]. DNA methyltransferases and their isoforms are expressed in a tissue- or cell-type-specific manner which suggests their participation in distinct developmental or differentiation process. In the liver, and to a lesser extent in lungs, DNMT3a and DNMT3b mRNA were reduced. Since these enzymes are crucial for de novo methylation [53] it suggests that this decrease could protect these organs from accumulating unwanted epigenetic modifications that could be triggered by environmental harshness, despite a strong deregulation of gene expression. These alterations would not be durably recorded, however, this observation does not preclude the possibility of a progressive passive demethylation. The overall activation in the placenta of genes involved in modulating chromatin structure in rather short delays and genes modifying chemically and covalently the DNA by methylation and thus for longer delays and heritable modifications, is much less problematic owing to the transient nature of the placenta. One peculiar category of genes regulated by epigenetic means is imprinted genes. We show here that these genes are statistically different from the rest of the genome in the kidney where there is an excess of upregulated imprinted genes, and to a lesser extent in the placenta where there is a lack of down-regulated imprinted genes. In the placenta, alterations of imprinting are known to impact normal function. For instance, it has recently been shown in mouse models that a partial reduction of Ascl2 expression results in severe structure anomalies of the placenta [54]. Similar observations were seen for Phlda2. In addition, numerous reports mention alterations of methylation and expression of H19 and IGF2 in IUGR [31], [55], [56]. Our own results reveal different imprinted genes that are altered at the expression level in the placenta, which may overall contribute to the deregulation of the ‘imprinted gene network’ at work in this organ [57].

In an attempt to define hierarchy of the factors that are modified in IUGR, we used a statistical approach aiming at identifying statistical differences between the occurrence of transcription factors binding sites according to the genes that are modified. This approach has previously be used successfully [10], [58]. In the additional organs studied here, only the liver revealed such a deviation between induced and repressed genes. Interestingly in this organ HNF4A binding sites were overrepresented in down-regulated genes. This transcription factor plays an important role in weight regulation and insulin signaling [59], especially by regulating apolipoprotein promoters [60]. It is thus tempting to suggest that in IUGR, deregulations of genes in the liver passes through this HNF4/fat/insulin pathway.

In summary of the present study, we can assume that gene alteration expression in the IUGR model contribute to the possibility of modifying long-term disease risk associated with unbalanced nutrition in fetal life.

## Supporting Information

Figure S1 Correlation between qPCR and microarray data for the comparison between IUGR and normal rat fetuses for lungs liver and heart. The log_2_ of the induction ratio for que qPCR was presented in abscissa and the log_2_ of the induction ratio for the microarray in ordinate. The coefficient of determination of the linear regression (R^2^) is given for each organ.
(PPTX)

Figure S2 Pathways identified by Genomatix as encompassing modified genes in the placenta; A = VEGFR1 specific signals, B = EGFR1 signalling, C = Ataxia telangectasia signaling pathway. Color code is function of the intensity of modification: blue for down-regulated genes, red for up-regulated genes, orange: not modified, and grey genes in the cascade but not modified in IUGR.
(PPTX)

Figure S3 Pathways encompassing genes modified in the heart in IUGR pups (color codes as in Figure S2). A: FOXA2 and FOXA3 networks; B: FOXA1 transcription factor network, C: Hedgehog signaling network. Below the table indicates the p-values for the enrichment.
(PPTX)

Figure S4 Pathways encompassing genes modified in the liver in IUGR pups (color codes as in Figure S2). A: Negative regulation of GDP-GTP exchange signaling, B: Heteromeric GPCR signaling pathway.
(PPTX)

Figure S5 Pathways encompassing genes modified in the lungs in IUGR pups (color codes as in Figure S2). A: Regulation of GDP-GTP exchange signaling, B: IL-7 signaling.
(PPTX)

Figure S6 Non-supervised hierarchical classification of gene expression in the various tissues analyzed in the study. The clustering correctly assembles the different tissues, and generally contrasts correctly the IUGR versus normal condition, except for lungs where the number of modified transcripts is so high (∼34%) that IUGR and normal are separated from all the other clusters.
(PPTX)

Table S1 List of epigenetic regulators and their degree of alterations by induced IUGR in the rat model.
(PPTX)

Table S2 List of imprinted genes and their degree of transcriptional modification by induced IUGR in the rat model.
(PPTX)

Table S3 Body weight, placenta, liver, lung, heart and kidney weights and number of nephrons in low protein group (LP) and in control pups group (C).
(PPTX)

Material S1 Organ per organ analysis of pathways enriched in modified genes in the various organs under scrutiny.
(DOC)
