# Supplementary figures and images for: The Intensity of IUGR-Induced Transcriptome Deregulations Is Inversely Correlated with the Onset of Organ Function in a Rat Model
Source: PLoS One. 2011 Jun 22;6(6):e21222. doi: 10.1371/journal.pone.0021222 (PMC3120850; doi:10.1371/journal.pone.0021222)

## Slide 1
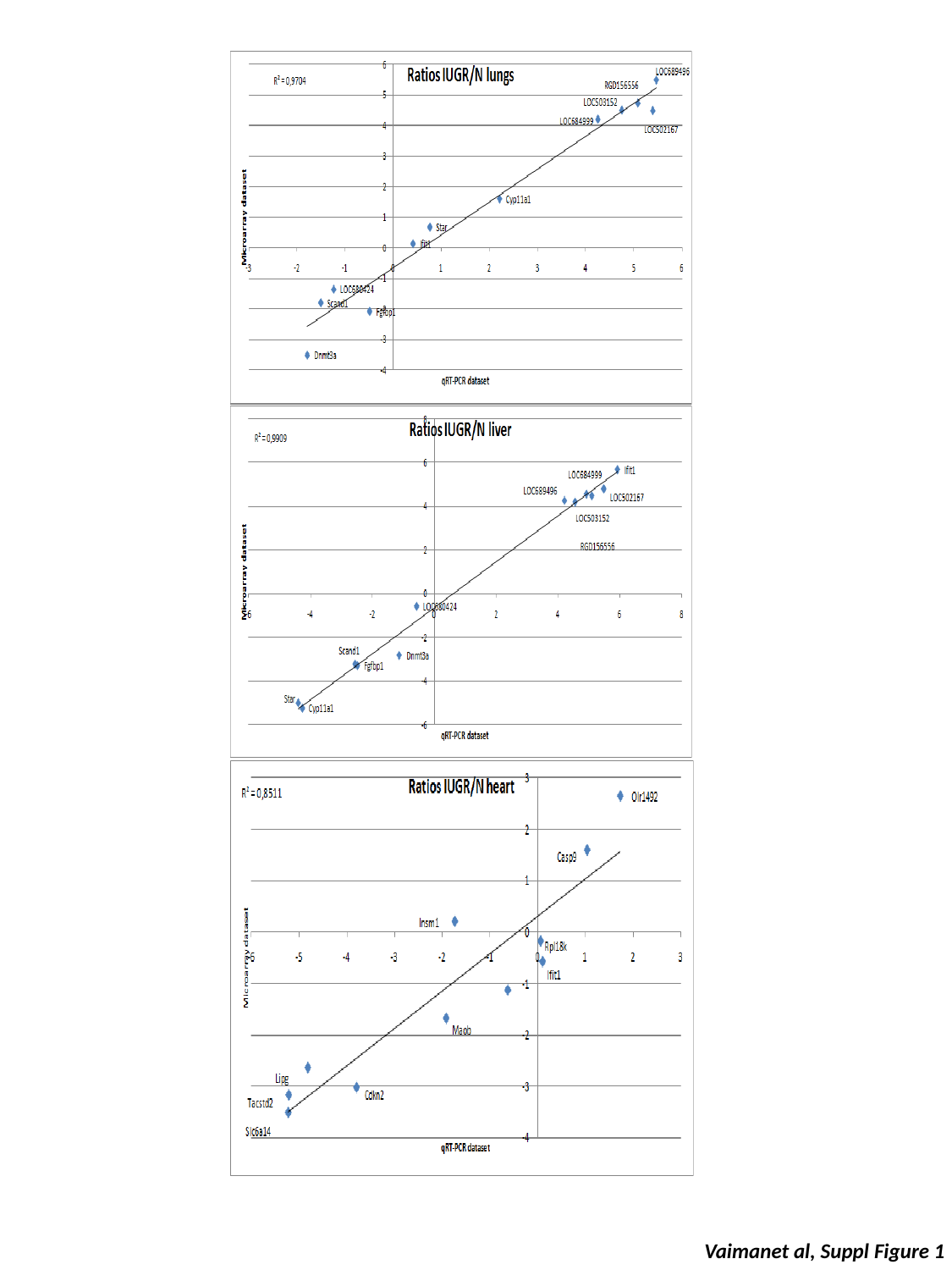

Vaimanet al, Suppl Figure 1

Supplement: Figure S1 — Correlation between qPCR and microarray data for the comparison between IUGR and normal rat fetuses for lungs liver and heart. The log2 of the induction ratio for que qPCR was presented in abscissa and the log2 of the induction ratio for the microarray in ordinate. The coefficient of determination of the linear regression (R2) is given for each organ. (PPTX) [file pone.0021222.s001.pptx]

## Slide 1
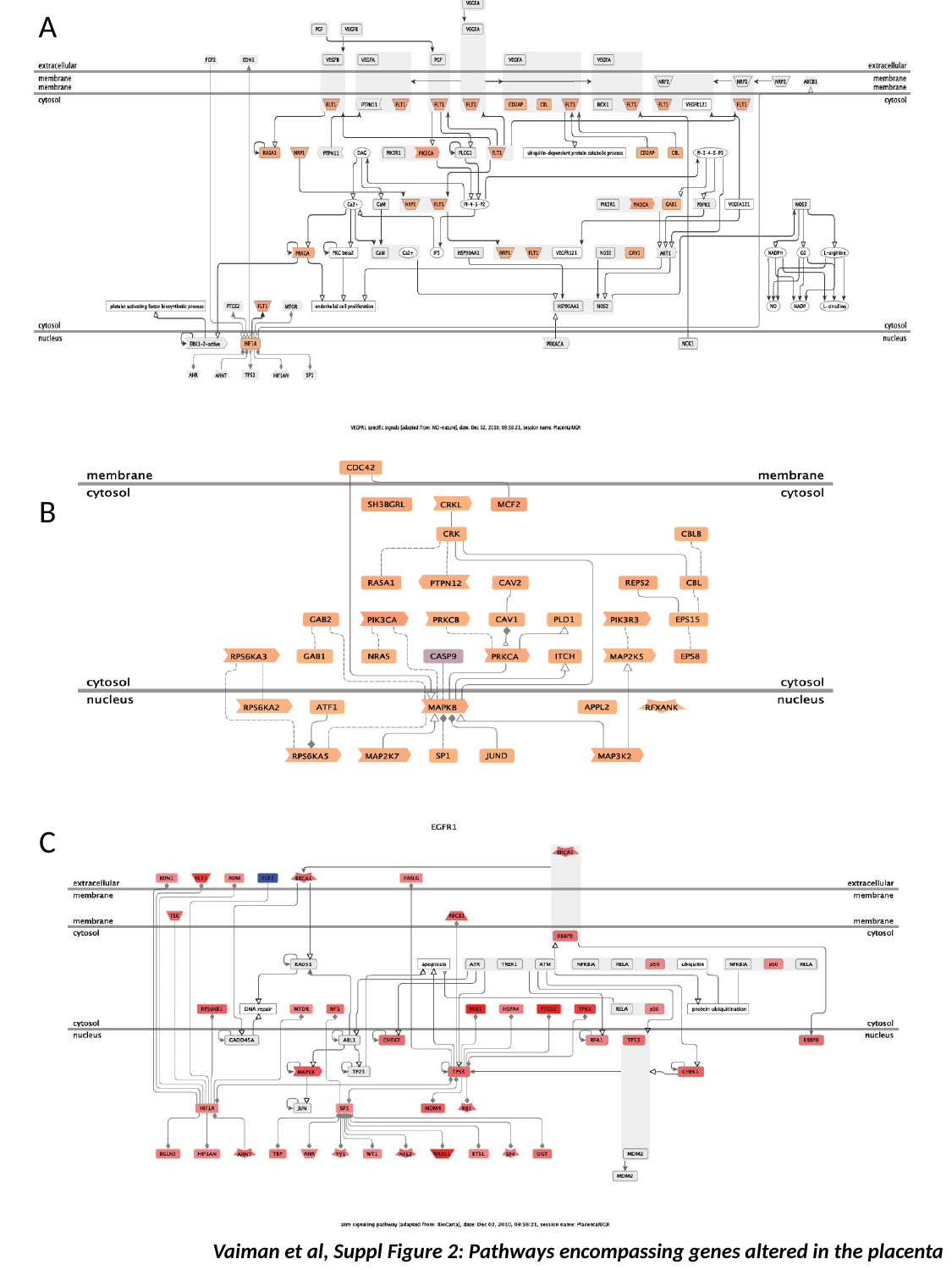

A
B
C
Vaiman et al, Suppl Figure 2: Pathways encompassing genes altered in the placenta

Supplement: Figure S2 — Pathways identified by Genomatix as encompassing modified genes in the placenta; A = VEGFR1 specific signals, B = EGFR1 signalling, C = Ataxia telangectasia signaling pathway. Color code is function of the intensity of modification: blue for down-regulated genes, red for up-regulated genes, orange: not modified, and grey genes in the cascade but not modified in IUGR. (PPTX) [file pone.0021222.s002.pptx]

## Slide 1
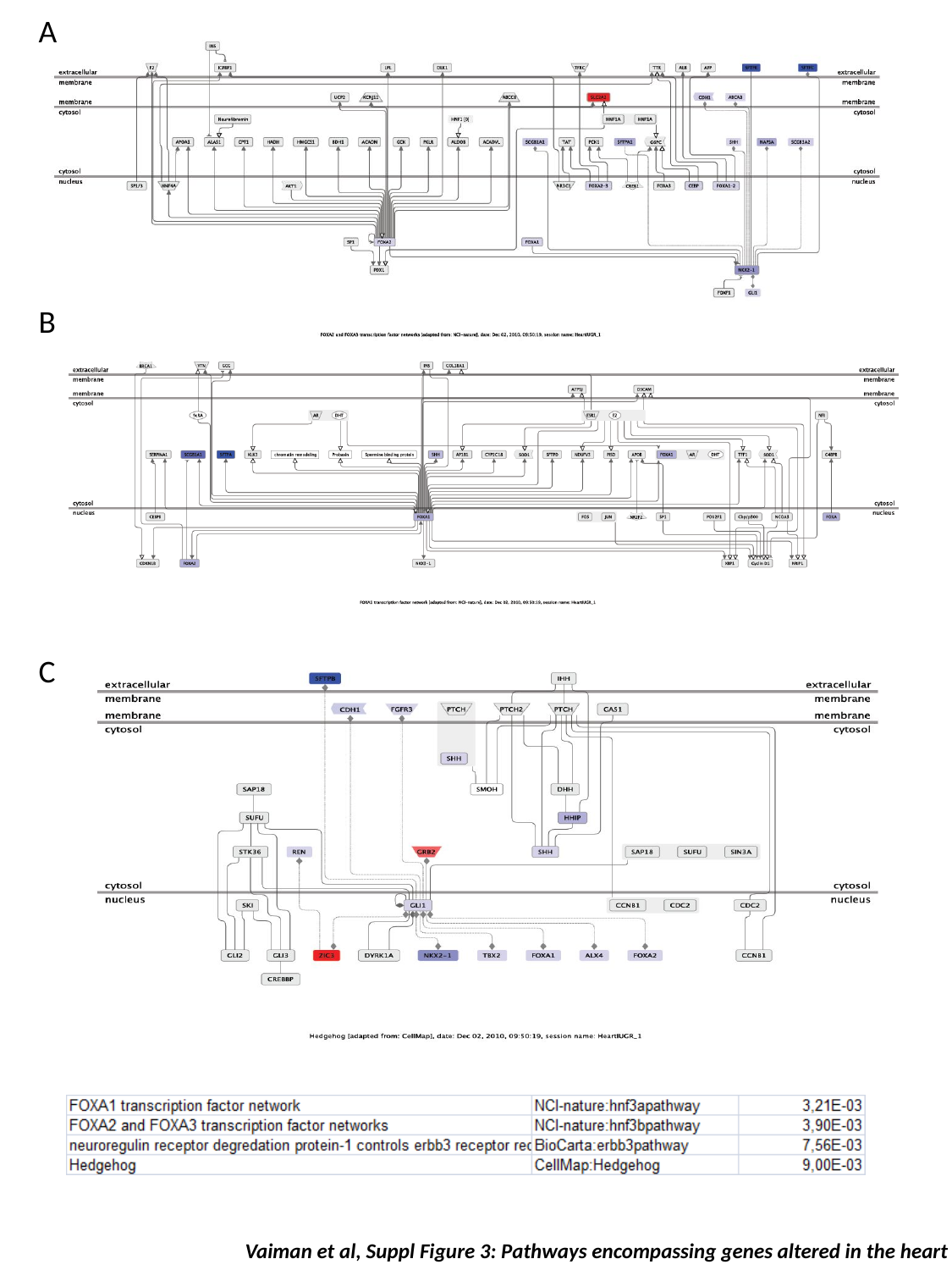

A
B
C
Vaiman et al, Suppl Figure 3: Pathways encompassing genes altered in the heart

Supplement: Figure S3 — Pathways encompassing genes modified in the heart in IUGR pups (color codes as in Figure S2). A: FOXA2 and FOXA3 networks; B: FOXA1 transcription factor network, C: Hedgehog signaling network. Below the table indicates the p-values for the enrichment. (PPTX) [file pone.0021222.s003.pptx]

## Slide 1
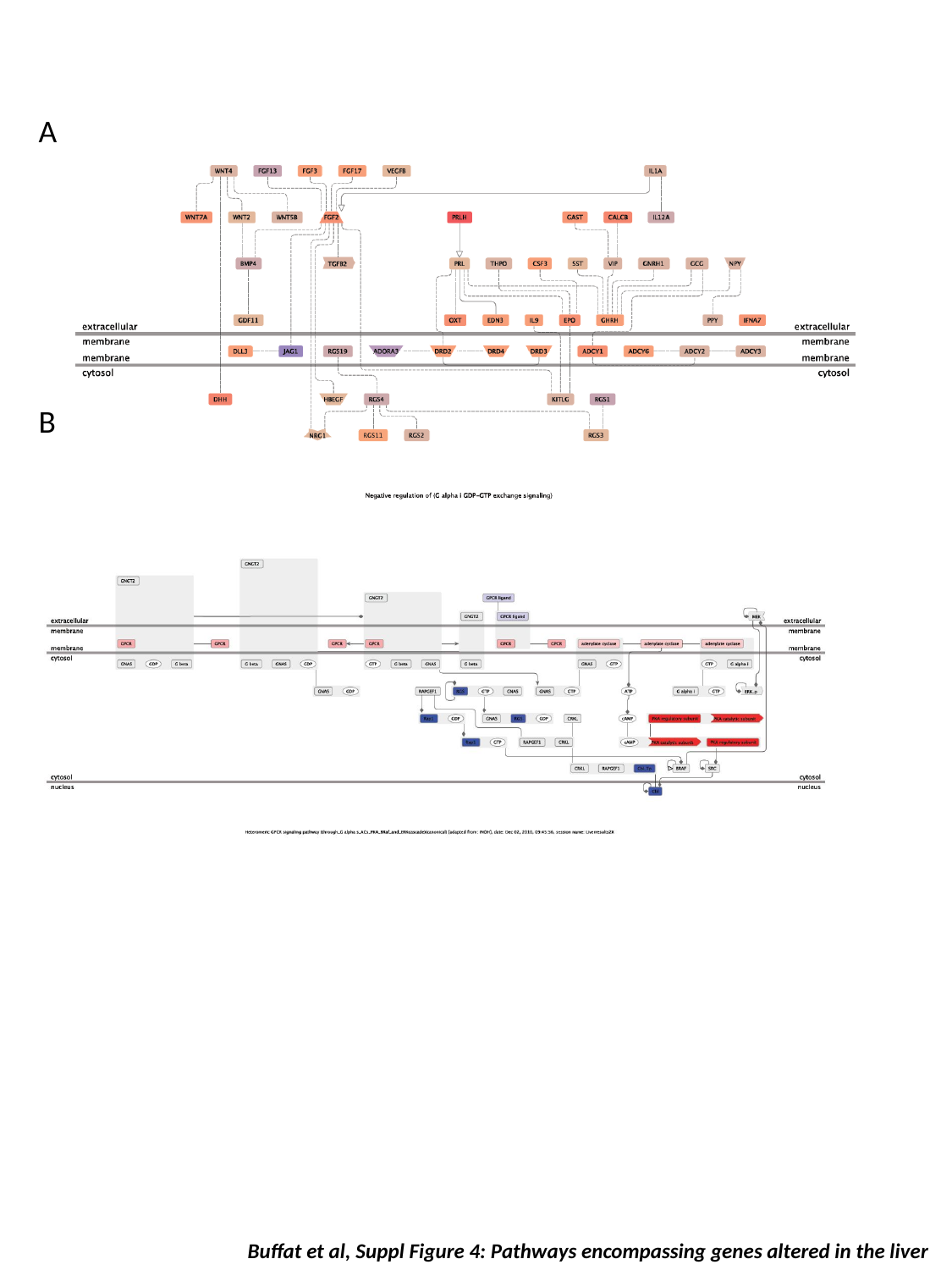

A
B
Buffat et al, Suppl Figure 4: Pathways encompassing genes altered in the liver

Supplement: Figure S4 — Pathways encompassing genes modified in the liver in IUGR pups (color codes as in Figure S2). A: Negative regulation of GDP-GTP exchange signaling, B: Heteromeric GPCR signaling pathway. (PPTX) [file pone.0021222.s004.pptx]

## Slide 1
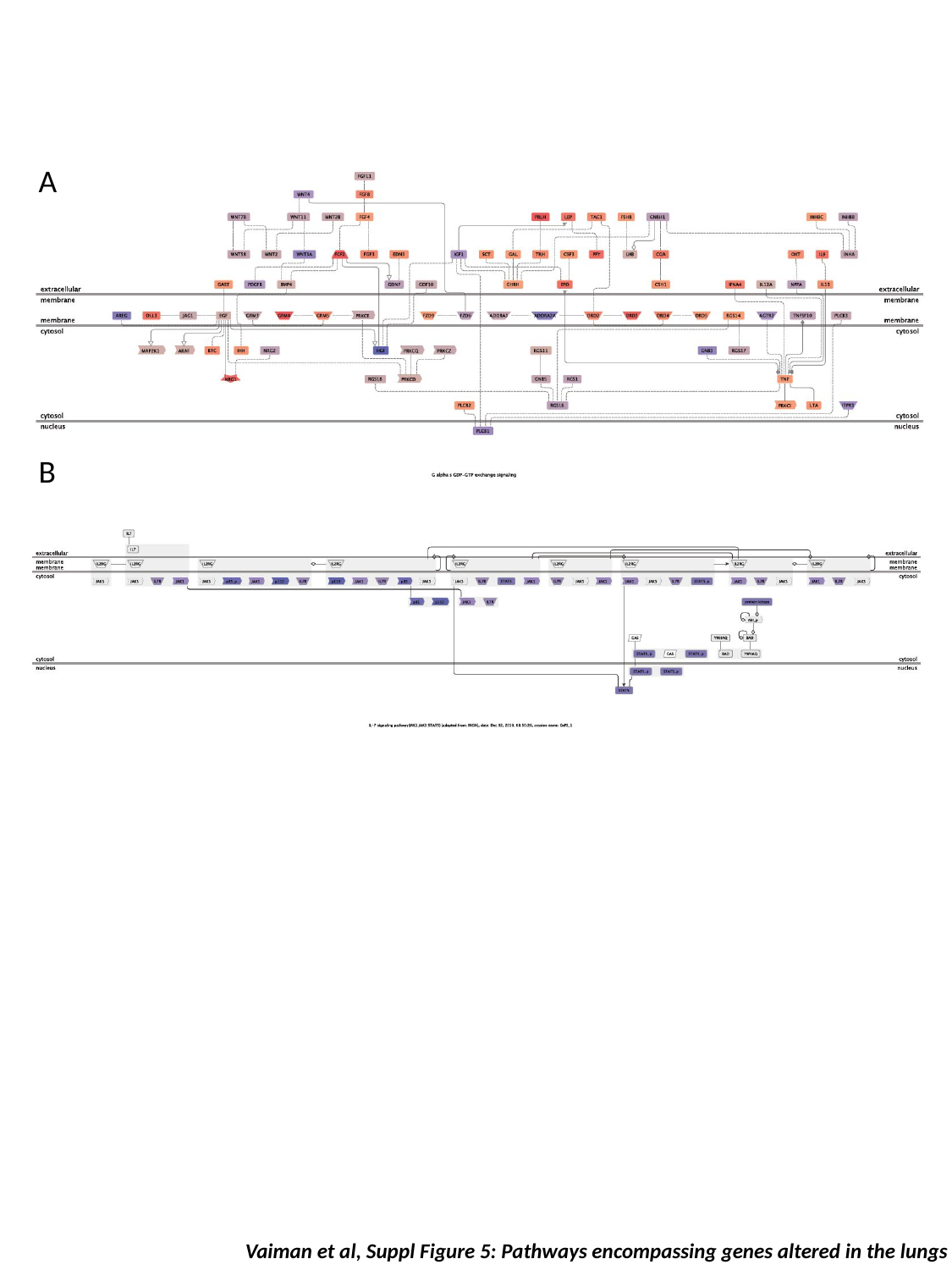

A
B
Vaiman et al, Suppl Figure 5: Pathways encompassing genes altered in the lungs

Supplement: Figure S5 — Pathways encompassing genes modified in the lungs in IUGR pups (color codes as in Figure S2). A: Regulation of GDP-GTP exchange signaling, B: IL-7 signaling. (PPTX) [file pone.0021222.s005.pptx]

## Slide 1
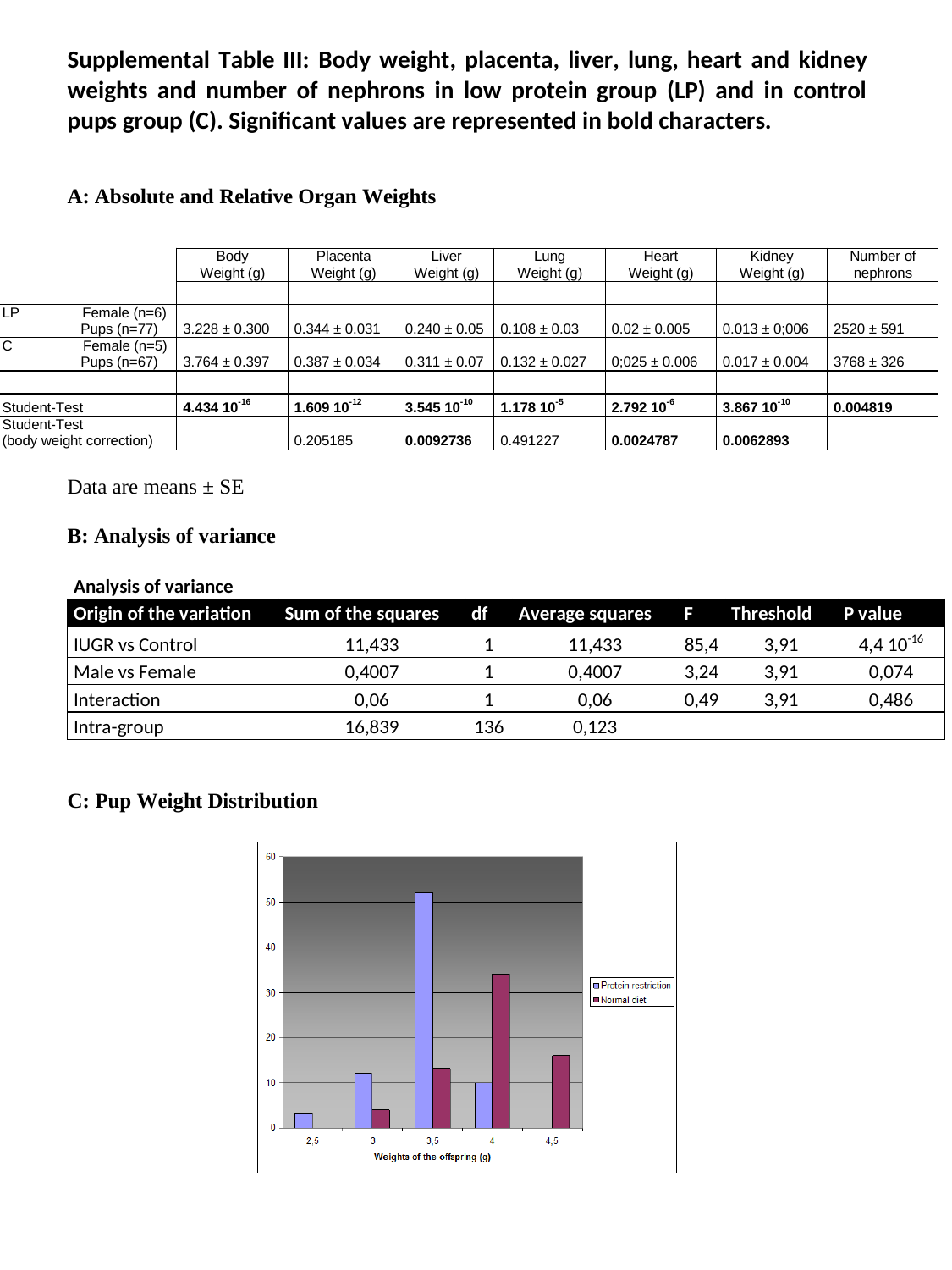

Supplement: Table S3 — Body weight, placenta, liver, lung, heart and kidney weights and number of nephrons in low protein group (LP) and in control pups group (C). (PPTX) [file pone.0021222.s009.pptx]
