# Supplementary material for: The Intensity of IUGR-Induced Transcriptome Deregulations Is Inversely Correlated with the Onset of Organ Function in a Rat Model
Source: PLoS One. 2011 Jun 22;6(6):e21222. doi: 10.1371/journal.pone.0021222 (PMC3120850; doi:10.1371/journal.pone.0021222)
Supplement: Figure S6 — Non-supervised hierarchical classification of gene expression in the various tissues analyzed in the study. The clustering correctly assembles the different tissues, and generally contrasts correctly the IUGR versus normal condition, except for lungs where the number of modified transcripts is so high (∼34%) that IUGR and normal are separated from all the other clusters. (PPTX) [file pone.0021222.s006.pptx]

## Slide 1
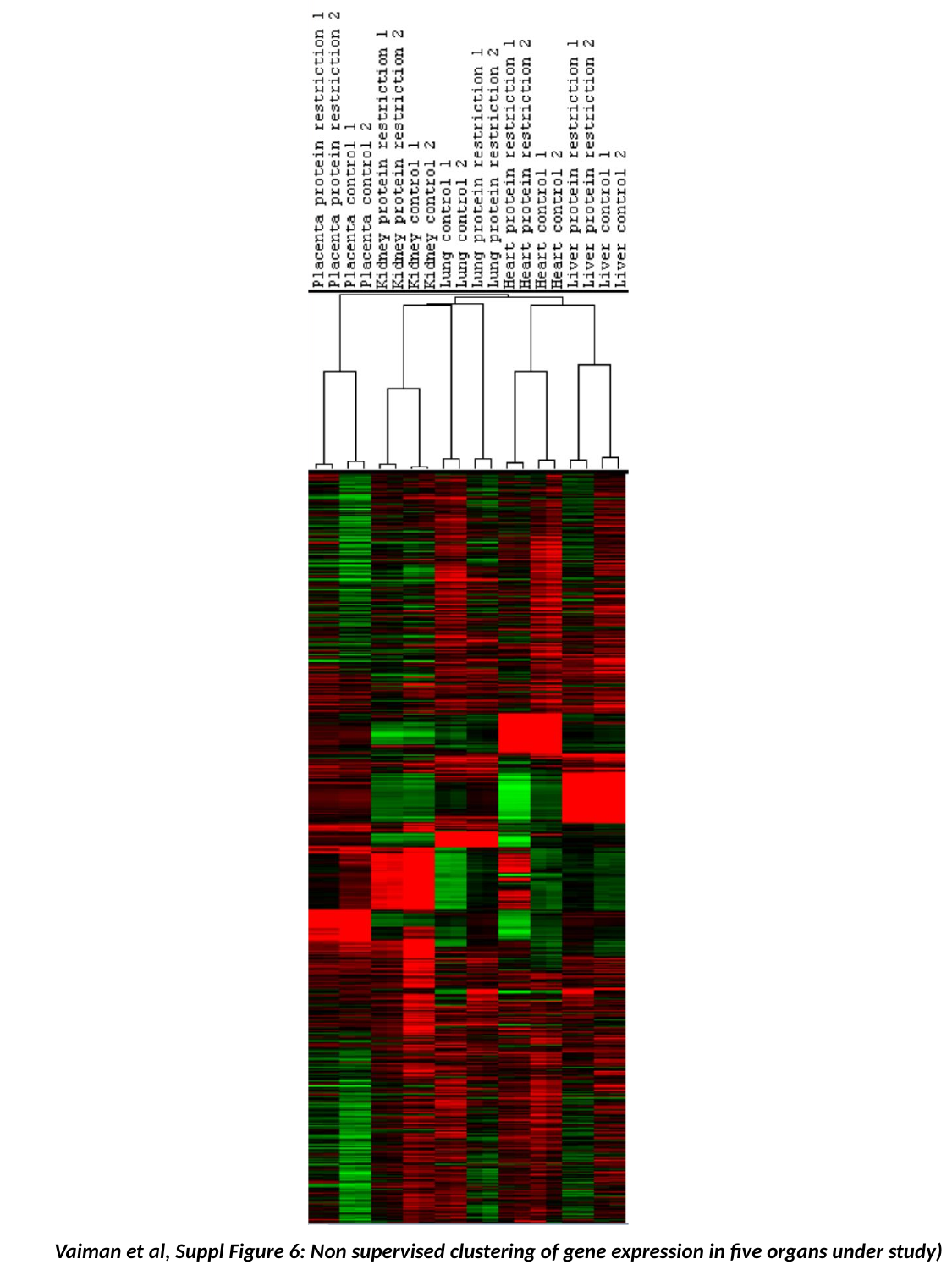

Vaiman et al, Suppl Figure 6: Non supervised clustering of gene expression in five organs under study)
