# Supplementary material for: The Intensity of IUGR-Induced Transcriptome Deregulations Is Inversely Correlated with the Onset of Organ Function in a Rat Model
Source: PLoS One. 2011 Jun 22;6(6):e21222. doi: 10.1371/journal.pone.0021222 (PMC3120850; doi:10.1371/journal.pone.0021222)
Supplement: Table S1 — List of epigenetic regulators and their degree of alterations by induced IUGR in the rat model. (PPTX) [file pone.0021222.s007.pptx]

## Slide 1
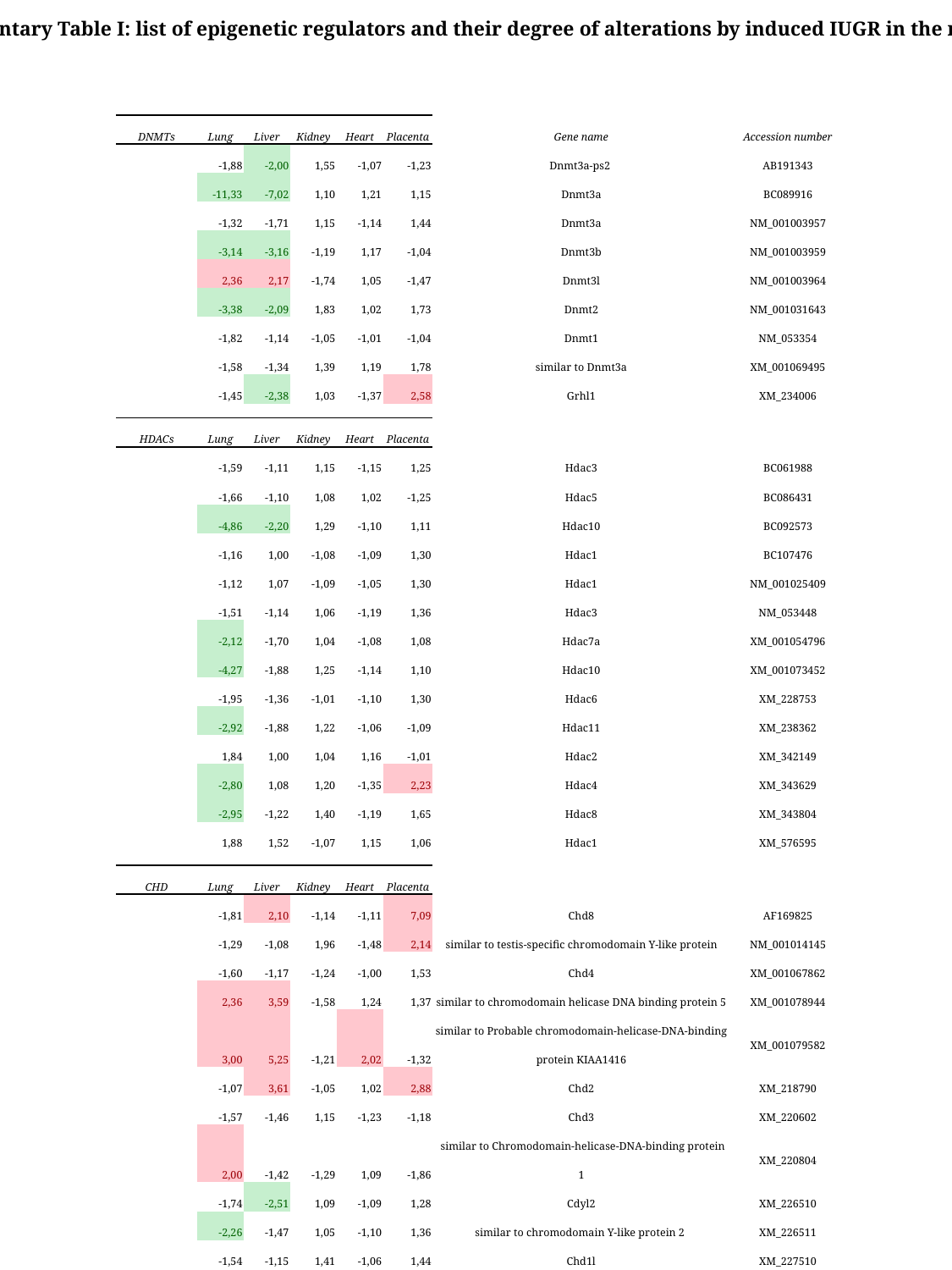

Supplementary Table I: list of epigenetic regulators and their degree of alterations by induced IUGR in the rat model.
| DNMTs | Lung | Liver | Kidney | Heart | Placenta | Gene name | Accession number |
| --- | --- | --- | --- | --- | --- | --- | --- |
| | -1,88 | -2,00 | 1,55 | -1,07 | -1,23 | Dnmt3a-ps2 | AB191343 |
| | -11,33 | -7,02 | 1,10 | 1,21 | 1,15 | Dnmt3a | BC089916 |
| | -1,32 | -1,71 | 1,15 | -1,14 | 1,44 | Dnmt3a | NM\_001003957 |
| | -3,14 | -3,16 | -1,19 | 1,17 | -1,04 | Dnmt3b | NM\_001003959 |
| | 2,36 | 2,17 | -1,74 | 1,05 | -1,47 | Dnmt3l | NM\_001003964 |
| | -3,38 | -2,09 | 1,83 | 1,02 | 1,73 | Dnmt2 | NM\_001031643 |
| | -1,82 | -1,14 | -1,05 | -1,01 | -1,04 | Dnmt1 | NM\_053354 |
| | -1,58 | -1,34 | 1,39 | 1,19 | 1,78 | similar to Dnmt3a | XM\_001069495 |
| | -1,45 | -2,38 | 1,03 | -1,37 | 2,58 | Grhl1 | XM\_234006 |
| | | | | | | | |
| HDACs | Lung | Liver | Kidney | Heart | Placenta | | |
| | -1,59 | -1,11 | 1,15 | -1,15 | 1,25 | Hdac3 | BC061988 |
| | -1,66 | -1,10 | 1,08 | 1,02 | -1,25 | Hdac5 | BC086431 |
| | -4,86 | -2,20 | 1,29 | -1,10 | 1,11 | Hdac10 | BC092573 |
| | -1,16 | 1,00 | -1,08 | -1,09 | 1,30 | Hdac1 | BC107476 |
| | -1,12 | 1,07 | -1,09 | -1,05 | 1,30 | Hdac1 | NM\_001025409 |
| | -1,51 | -1,14 | 1,06 | -1,19 | 1,36 | Hdac3 | NM\_053448 |
| | -2,12 | -1,70 | 1,04 | -1,08 | 1,08 | Hdac7a | XM\_001054796 |
| | -4,27 | -1,88 | 1,25 | -1,14 | 1,10 | Hdac10 | XM\_001073452 |
| | -1,95 | -1,36 | -1,01 | -1,10 | 1,30 | Hdac6 | XM\_228753 |
| | -2,92 | -1,88 | 1,22 | -1,06 | -1,09 | Hdac11 | XM\_238362 |
| | 1,84 | 1,00 | 1,04 | 1,16 | -1,01 | Hdac2 | XM\_342149 |
| | -2,80 | 1,08 | 1,20 | -1,35 | 2,23 | Hdac4 | XM\_343629 |
| | -2,95 | -1,22 | 1,40 | -1,19 | 1,65 | Hdac8 | XM\_343804 |
| | 1,88 | 1,52 | -1,07 | 1,15 | 1,06 | Hdac1 | XM\_576595 |
| | | | | | | | |
| CHD | Lung | Liver | Kidney | Heart | Placenta | | |
| | -1,81 | 2,10 | -1,14 | -1,11 | 7,09 | Chd8 | AF169825 |
| | -1,29 | -1,08 | 1,96 | -1,48 | 2,14 | similar to testis-specific chromodomain Y-like protein | NM\_001014145 |
| | -1,60 | -1,17 | -1,24 | -1,00 | 1,53 | Chd4 | XM\_001067862 |
| | 2,36 | 3,59 | -1,58 | 1,24 | 1,37 | similar to chromodomain helicase DNA binding protein 5 | XM\_001078944 |
| | 3,00 | 5,25 | -1,21 | 2,02 | -1,32 | similar to Probable chromodomain-helicase-DNA-binding protein KIAA1416 | XM\_001079582 |
| | -1,07 | 3,61 | -1,05 | 1,02 | 2,88 | Chd2 | XM\_218790 |
| | -1,57 | -1,46 | 1,15 | -1,23 | -1,18 | Chd3 | XM\_220602 |
| | 2,00 | -1,42 | -1,29 | 1,09 | -1,86 | similar to Chromodomain-helicase-DNA-binding protein 1 | XM\_220804 |
| | -1,74 | -2,51 | 1,09 | -1,09 | 1,28 | Cdyl2 | XM\_226510 |
| | -2,26 | -1,47 | 1,05 | -1,10 | 1,36 | similar to chromodomain Y-like protein 2 | XM\_226511 |
| | -1,54 | -1,15 | 1,41 | -1,06 | 1,44 | Chd1l | XM\_227510 |
| | -1,26 | -1,20 | 1,14 | 1,07 | -1,01 | Chd6 | XM\_230814 |
| | -2,37 | -1,44 | -1,07 | -1,21 | 1,04 | similar to chromodomain protein, Y chromosome-like isoform a | XM\_231456 |
| | -3,40 | -1,38 | 1,07 | -1,17 | 1,94 | Chd7 | XM\_232671 |
| | -1,06 | 2,98 | -1,23 | 1,11 | 7,99 | Chd1 | XM\_238731 |
| | -1,18 | -1,13 | -1,09 | 1,14 | 1,55 | Chd8 | XM\_573762 |
| | | | | | | | |
| BRD | Lung | Liver | Kidney | Heart | Placenta | | |
| | -1,79 | -1,18 | -1,94 | 1,85 | -1,83 | Brdt | BC078999 |
| | -1,91 | -1,62 | 1,31 | -1,02 | 1,62 | Brd8 | NM\_001008509 |
| | -1,18 | -1,05 | -1,06 | -1,02 | 1,15 | Brd2 | NM\_212495 |
| | -1,38 | -1,15 | 1,02 | 1,03 | 1,28 | similar to bromodomain and PHD finger-containing protein 1 isoform 1 | XM\_001054156 |
| | -1,09 | -2,05 | 1,38 | 1,19 | 1,95 | Brd4 | XM\_001065925 |
| | -1,69 | -1,21 | 1,25 | -1,08 | 1,51 | Brd9 | XM\_217740 |
| | -2,10 | -1,54 | 1,45 | -1,15 | 2,85 | similar to bromodomain and WD repeat domain containing 2 | XM\_219377 |
| | -1,13 | 1,04 | 1,13 | -1,02 | 1,79 | Brwd1 | XM\_221627 |
| | -2,64 | -1,50 | -1,33 | -1,05 | 1,97 | Baz2a | XM\_222315 |
| | -3,20 | -1,15 | 1,13 | -1,22 | 1,19 | Brpf3 | XM\_228039 |
| | -2,92 | -1,22 | 1,15 | -1,05 | 3,25 | Brwd3 | XM\_228518 |
| | -1,94 | -2,35 | 1,75 | 1,11 | 4,69 | Baz2b | XM\_229225 |
| | -1,58 | -1,33 | -1,03 | -1,04 | 2,08 | Baz1a | XM\_234156 |
| | -2,66 | -1,08 | -1,01 | -1,14 | 1,67 | Brd1 | XM\_235552 |
| | -1,02 | -1,20 | 1,16 | -1,03 | 1,29 | Brd7 | XM\_341653 |
| | -1,10 | -1,25 | 1,53 | 1,11 | 1,28 | Brd3 | XM\_342396 |
| | -1,45 | -1,12 | 1,01 | -1,01 | 1,30 | Baz1b | XM\_347166 |
| | 3,55 | 1,50 | -1,19 | 1,15 | 1,07 | Brdt | XM\_573544 |
| | | | | | | | |
| Histone methyl/acetyl transferase | Lung | Liver | Kidney | Heart | Placenta | | |
| | -1,23 | 2,00 | -1,04 | -1,07 | 2,56 | Myst2 | AY241457 |
| | 1,69 | 1,02 | 1,39 | 1,19 | 1,69 | Hat1 | NM\_001009657 |
| | -1,63 | -1,10 | 1,31 | -1,00 | 1,67 | Myst1 | NM\_001017378 |
| | -1,31 | -1,29 | 1,06 | -1,08 | 1,48 | Myst2 | NM\_181081 |
| | -1,74 | -1,19 | 1,15 | -1,22 | -1,01 | Ehmt2 | NM\_212463 |
| | -1,88 | -1,16 | 1,12 | 1,04 | 1,91 | similar to Histone acetyltransferase MYST4 | XM\_001067699 |
| | 1,66 | 3,64 | -1,33 | 1,21 | -1,20 | similar to Histone-lysine N-methyltransferase, H3 lysine-9 specific 4 | XM\_001072340 |
| | 1,14 | 2,86 | -1,15 | -1,15 | 4,09 | similar to Histone-lysine N-methyltransferase, H3 lysine-9 specific 4 | XM\_001072375 |
| | -3,22 | -1,72 | -1,03 | -1,26 | 1,59 | similar to Histone-lysine N-methyltransferase, H3 lysine-4 specific SET7 | XM\_001072672 |
| | -1,16 | 1,45 | 1,03 | 1,03 | 1,72 | Myst3 | XM\_225008 |
| | -2,21 | 1,00 | -1,01 | -1,05 | 1,46 | Ehmt1 | XM\_342379 |
| | -2,34 | -1,15 | -1,08 | -1,09 | 1,42 | Dot1l | XM\_343159 |
