# Supplementary material for: The Intensity of IUGR-Induced Transcriptome Deregulations Is Inversely Correlated with the Onset of Organ Function in a Rat Model
Source: PLoS One. 2011 Jun 22;6(6):e21222. doi: 10.1371/journal.pone.0021222 (PMC3120850; doi:10.1371/journal.pone.0021222)
Supplement: Table S2 — List of imprinted genes and their degree of transcriptional modification by induced IUGR in the rat model. (PPTX) [file pone.0021222.s008.pptx]

## Slide 1
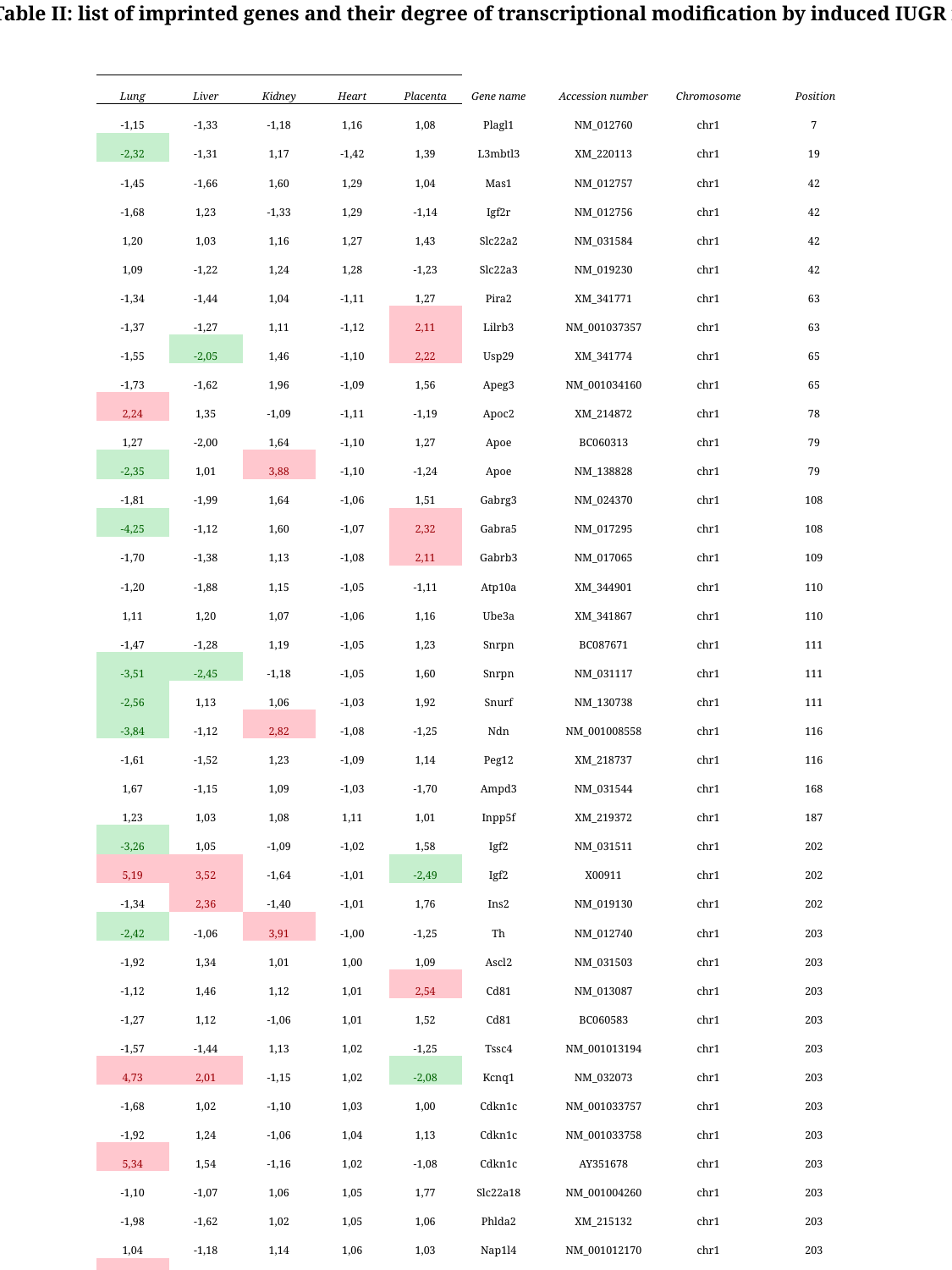

Supplementary Table II: list of imprinted genes and their degree of transcriptional modification by induced IUGR in the rat model.
| Lung | Liver | Kidney | Heart | Placenta | Gene name | Accession number | Chromosome | Position |
| --- | --- | --- | --- | --- | --- | --- | --- | --- |
| -1,15 | -1,33 | -1,18 | 1,16 | 1,08 | Plagl1 | NM\_012760 | chr1 | 7 |
| -2,32 | -1,31 | 1,17 | -1,42 | 1,39 | L3mbtl3 | XM\_220113 | chr1 | 19 |
| -1,45 | -1,66 | 1,60 | 1,29 | 1,04 | Mas1 | NM\_012757 | chr1 | 42 |
| -1,68 | 1,23 | -1,33 | 1,29 | -1,14 | Igf2r | NM\_012756 | chr1 | 42 |
| 1,20 | 1,03 | 1,16 | 1,27 | 1,43 | Slc22a2 | NM\_031584 | chr1 | 42 |
| 1,09 | -1,22 | 1,24 | 1,28 | -1,23 | Slc22a3 | NM\_019230 | chr1 | 42 |
| -1,34 | -1,44 | 1,04 | -1,11 | 1,27 | Pira2 | XM\_341771 | chr1 | 63 |
| -1,37 | -1,27 | 1,11 | -1,12 | 2,11 | Lilrb3 | NM\_001037357 | chr1 | 63 |
| -1,55 | -2,05 | 1,46 | -1,10 | 2,22 | Usp29 | XM\_341774 | chr1 | 65 |
| -1,73 | -1,62 | 1,96 | -1,09 | 1,56 | Apeg3 | NM\_001034160 | chr1 | 65 |
| 2,24 | 1,35 | -1,09 | -1,11 | -1,19 | Apoc2 | XM\_214872 | chr1 | 78 |
| 1,27 | -2,00 | 1,64 | -1,10 | 1,27 | Apoe | BC060313 | chr1 | 79 |
| -2,35 | 1,01 | 3,88 | -1,10 | -1,24 | Apoe | NM\_138828 | chr1 | 79 |
| -1,81 | -1,99 | 1,64 | -1,06 | 1,51 | Gabrg3 | NM\_024370 | chr1 | 108 |
| -4,25 | -1,12 | 1,60 | -1,07 | 2,32 | Gabra5 | NM\_017295 | chr1 | 108 |
| -1,70 | -1,38 | 1,13 | -1,08 | 2,11 | Gabrb3 | NM\_017065 | chr1 | 109 |
| -1,20 | -1,88 | 1,15 | -1,05 | -1,11 | Atp10a | XM\_344901 | chr1 | 110 |
| 1,11 | 1,20 | 1,07 | -1,06 | 1,16 | Ube3a | XM\_341867 | chr1 | 110 |
| -1,47 | -1,28 | 1,19 | -1,05 | 1,23 | Snrpn | BC087671 | chr1 | 111 |
| -3,51 | -2,45 | -1,18 | -1,05 | 1,60 | Snrpn | NM\_031117 | chr1 | 111 |
| -2,56 | 1,13 | 1,06 | -1,03 | 1,92 | Snurf | NM\_130738 | chr1 | 111 |
| -3,84 | -1,12 | 2,82 | -1,08 | -1,25 | Ndn | NM\_001008558 | chr1 | 116 |
| -1,61 | -1,52 | 1,23 | -1,09 | 1,14 | Peg12 | XM\_218737 | chr1 | 116 |
| 1,67 | -1,15 | 1,09 | -1,03 | -1,70 | Ampd3 | NM\_031544 | chr1 | 168 |
| 1,23 | 1,03 | 1,08 | 1,11 | 1,01 | Inpp5f | XM\_219372 | chr1 | 187 |
| -3,26 | 1,05 | -1,09 | -1,02 | 1,58 | Igf2 | NM\_031511 | chr1 | 202 |
| 5,19 | 3,52 | -1,64 | -1,01 | -2,49 | Igf2 | X00911 | chr1 | 202 |
| -1,34 | 2,36 | -1,40 | -1,01 | 1,76 | Ins2 | NM\_019130 | chr1 | 202 |
| -2,42 | -1,06 | 3,91 | -1,00 | -1,25 | Th | NM\_012740 | chr1 | 203 |
| -1,92 | 1,34 | 1,01 | 1,00 | 1,09 | Ascl2 | NM\_031503 | chr1 | 203 |
| -1,12 | 1,46 | 1,12 | 1,01 | 2,54 | Cd81 | NM\_013087 | chr1 | 203 |
| -1,27 | 1,12 | -1,06 | 1,01 | 1,52 | Cd81 | BC060583 | chr1 | 203 |
| -1,57 | -1,44 | 1,13 | 1,02 | -1,25 | Tssc4 | NM\_001013194 | chr1 | 203 |
| 4,73 | 2,01 | -1,15 | 1,02 | -2,08 | Kcnq1 | NM\_032073 | chr1 | 203 |
| -1,68 | 1,02 | -1,10 | 1,03 | 1,00 | Cdkn1c | NM\_001033757 | chr1 | 203 |
| -1,92 | 1,24 | -1,06 | 1,04 | 1,13 | Cdkn1c | NM\_001033758 | chr1 | 203 |
| 5,34 | 1,54 | -1,16 | 1,02 | -1,08 | Cdkn1c | AY351678 | chr1 | 203 |
| -1,10 | -1,07 | 1,06 | 1,05 | 1,77 | Slc22a18 | NM\_001004260 | chr1 | 203 |
| -1,98 | -1,62 | 1,02 | 1,05 | 1,06 | Phlda2 | XM\_215132 | chr1 | 203 |
| 1,04 | -1,18 | 1,14 | 1,06 | 1,03 | Nap1l4 | NM\_001012170 | chr1 | 203 |
| 3,05 | 1,23 | 1,60 | 1,11 | 1,10 | Osbpl5 | XM\_001066269 | chr1 | 204 |
| -1,25 | 1,67 | 1,64 | 1,09 | 1,41 | Osbpl5 | BC091326 | chr1 | 204 |
| -3,68 | 1,12 | -1,05 | 1,11 | 1,42 | Dhcr7 | NM\_022389 | chr1 | 204 |
| 1,00 | -1,62 | 1,48 | 1,30 | -1,60 | Ins1 | NM\_019129 | chr1 | 258 |
| -1,54 | -1,23 | 1,33 | -1,31 | 1,42 | Gatm | XM\_575224 | chr3 | 109 |
| -1,58 | -1,48 | 1,07 | -1,35 | 1,11 | Gatm | BC081785 | chr3 | 109 |
| -2,18 | 1,59 | 1,03 | -1,26 | 3,08 | Pax1 | XM\_230663 | chr3 | 136 |
| 3,41 | 1,91 | -1,41 | -1,25 | -1,78 | H13 | XM\_230734 | chr3 | 143 |
| -1,61 | 1,48 | -1,18 | -1,23 | -1,60 | Agouti | NM\_052979 | chr3 | 145 |
| -1,46 | -1,55 | 1,18 | -1,25 | 1,26 | Nnat | NM\_053601 | chr3 | 148 |
| -1,74 | -1,18 | 4,58 | -1,25 | -1,81 | Nnat | NM\_181687 | chr3 | 148 |
| -2,15 | -2,50 | 2,62 | -1,56 | 5,37 | Gnas | XM\_575296 | chr3 | 165 |
| -1,73 | -2,02 | -1,04 | -1,96 | 1,00 | Gnas | AF105254 | chr3 | 165 |
| -1,11 | -1,39 | 1,45 | -1,12 | -1,18 | Calcr | NM\_001034015 | chr4 | 28 |
| -1,22 | -1,31 | 1,27 | -1,12 | -1,05 | Calcr | L14618 | chr4 | 28 |
| -2,15 | -1,74 | 1,19 | -1,15 | 1,24 | Tfpi2 | NM\_173141 | chr4 | 28 |
| 2,42 | 2,22 | -1,62 | -1,23 | -1,70 | Ppp1r9a | NM\_053473 | chr4 | 29 |
| -1,64 | -1,25 | 1,10 | -1,21 | 2,44 | Pon1 | BC091403 | chr4 | 29 |
| -1,05 | 1,39 | 1,08 | -1,20 | 1,89 | Pon1 | NM\_032077 | chr4 | 29 |
| -3,34 | -1,63 | 1,15 | -1,19 | 1,06 | Pon3 | NM\_001004086 | chr4 | 30 |
| -1,23 | -1,36 | 1,26 | -1,13 | -1,04 | Pon2 | NM\_001013082 | chr4 | 30 |
| 2,12 | 1,79 | -1,67 | -1,13 | -1,99 | Dlx5 | NM\_012943 | chr4 | 31 |
| -4,04 | -1,38 | 2,12 | -1,12 | -1,31 | Mest | NM\_001009617 | chr4 | 186 |
| 6,10 | 2,31 | -2,37 | 1,19 | -2,99 | Dlk1 | NM\_053744 | chr6 | 134 |
| -3,10 | -2,16 | -1,30 | 1,16 | -1,89 | Dcn | NM\_024129 | chr7 | 35 |
| 2,10 | -1,01 | -1,29 | 1,27 | -1,20 | Kcnk9 | NM\_053405 | chr7 | 110 |
| 1,03 | -1,16 | 1,99 | -1,53 | -1,13 | L3mbtl2 | NM\_001033695 | chr7 | 120 |
| 3,40 | -1,04 | 1,10 | 1,27 | -1,28 | Slc38a4 | BC097292 | chr7 | 135 |
| -1,19 | -2,05 | 1,26 | 1,14 | -1,95 | Rasgrf1 | XM\_236468 | chr8 | 94 |
| -1,10 | -1,32 | 1,25 | 1,30 | 1,16 | Axin1 | NM\_024405 | chr10 | 15 |
| -4,32 | -2,63 | 1,04 | -1,23 | 3,41 | Ppp1cc | BC078825 | chr12 | 35 |
| -1,40 | 2,30 | -1,19 | -1,23 | 1,01 | Ppp1cc | NM\_022498 | chr12 | 35 |
| 1,61 | -1,02 | -1,05 | 1,17 | 1,27 | Ddc | NM\_012545 | chr14 | 92 |
| -1,93 | -1,17 | 1,31 | 1,17 | 1,79 | Ddc | U31884 | chr14 | 92 |
| -2,54 | -1,42 | 1,70 | 1,18 | -1,47 | similar to Grb10 protein | XM\_573664 | chr14 | 92 |
| 1,44 | -1,04 | 1,40 | 1,23 | 1,83 | Rb1 | XM\_344434 | chr15 | 53 |
| -1,82 | -1,90 | 1,05 | 1,24 | 1,67 | Htr2a | NM\_017254 | chr15 | 55 |
| -1,26 | -1,18 | 3,48 | -6,59 | 1,41 | Sfmbt2 | XM\_225556 | chr17 | 79 |
| 6,55 | 1,92 | -2,10 | 1,75 | -2,01 | Dmd | X69767 | chrX | 71 |
| -1,20 | -1,19 | 1,17 | 1,35 | 2,83 | Dmd | NM\_001005244 | chrX | 71 |
| -1,40 | -1,59 | -1,63 | 1,43 | -1,08 | Dmd | NM\_001005246 | chrX | 71 |
| -1,83 | 1,04 | -1,26 | 1,71 | 1,44 | Dmd | NM\_012698 | chrX | 71 |
| -1,36 | -1,18 | 1,02 | 1,33 | -1,23 | Dmd | AY326949 | chrX | 71 |
