# Supplementary material for: The Intensity of IUGR-Induced Transcriptome Deregulations Is Inversely Correlated with the Onset of Organ Function in a Rat Model
Source: PLoS One. 2011 Jun 22;6(6):e21222. doi: 10.1371/journal.pone.0021222 (PMC3120850; doi:10.1371/journal.pone.0021222)
Supplement: Material S1 — Organ per organ analysis of pathways enriched in modified genes in the various organs under scrutiny. (DOC) [file pone.0021222.s010.doc]

**SUPPLEMENTARY MATERIAL**

**Organ per organ analysis of pathways enriched in modified genes in the various organs under scrutiny**

**In the placenta**

Using Genomatix GePS, we identified five pathways enriched in modified genes: Reelin signaling, cdk regulation of DNA replication, Ataxia Telangectasia pathway, VEGFR1 and EGFR1 pathways, with p values ranging from 0.003 to 0.008. The VEGFR1 and EGFR1 pathways are presented (Supplemental Figures 2A and 2B). Both pathways encompass exclusively overexpressed genes, except caspase 9 which is down-regulated 2.3 fold. The induction of the VEGFR1 genes in the IUGR placenta is relevant since it suggests concerted attempts of the placenta to respond to the protein depletion by an improved angiogenesis. Various important ligands in the placental physiology such as the various isoforms of VEGF and the PGF are passing through the VEGFR1 cascade and the increase of this gene itself (the FLT1 gene encoding VEGFR1 being transcriptionnally induced 4.03 fold in our dataset) suggests a strong compensation mechanism towards vascularization of the placenta. The EGFR1 cascade is involved in the activation of the RAS and MAP kinases pathways, pertaining to cell proliferation, apoptosis and cell cycle. Concerning the ATM pathway, the ATM gene itself is not modified (Supplementary Figure 2C). The overall activation of this cascade (genes presented in red) shows an activation of tumor suppressor genes (TP53, BRCA) and checkpoint factors (CHEK1). This activation is probably consistent with a slowing in cell cycle progression. The fourth gene network significantly modified in the placenta encompasses genes involved in DNA replication controlled by various cyclin dependent kinases (CDK, not shown). An important number of genes involved in this pathway is significantly induced at the expressional level, amongst which various components of a transmembrane complex, and of two intracytoplasmic complexes involved in the control of DNA replication. This includes the induction of genes of the Origin Replication Complex (ORC) such as Cdc6, Cdt1 and mini-chromosome complex (MCM5, MCM6). This suggests an attempt at increasing mitosis efficiency in the IUGR placenta. A last group of genes found clusterized in the GePS analysis in the placenta is the reelin pathway, but the meaning of this group is elusive since this pathway is mainly known to be involved in brain function.

**4.2 In the heart**

In this organ, four pathways were significantly enriched: the FOXA1, FOXA2 and FOXA3 pathways, the neuroregulin degradation pathway, and the Hedgehog pathway, with p values ranging from 0.003 to 0.009. The induction of the FOXA2/FOXA3 pathway is consistent with their known involvement in starvation effects although the finding of a leading role in heart is quite new. The FOXA1 and FOXA2 cascades are intimately connected since FOXA1 is a well known transcriptional inducer of FOXA2 (Supplementary figures 3A and 3B). These genes appear down-regulated in the heart in IUGR (FOXA1 and FOXA2 reduced 2.72 and 2.73 fold, similar to the kidney and lungs respectively, while FOXA3 was not modified in heart but induced in the kidney), suggesting that the shortage in aminoacids in the heart is efficiently counterbalanced to preserve its normal function. These alterations are connected with the modification of the Sonic Hedgehog (SHH) pathway, which encompasses both FOXA1 and FOXA2 (Supplementary Figure 3C). The neuroregulin cascade, finally, appears strongly down-regulated. The biological significance of this deregulation remains elusive.

**4.3 In the kidney**

In this organ, 8 pathways were significant at the defined threshold. The three first pathways (Intrinsic prothrombin activation, extrinsinc prothrombin activation, and fibrinolysis pathways), encompass a considerably higher number of genes than expected (p values ranging from 4.6 10^-13^ to 1.5 10^-7^). The five other pathways (See Table 4A) had significance values more comparable to the one found in the other organs (from 0.001 to 0.007). These analyses of kidney gene deregulations are in perfect concordance with the analysis carried out in 2007, using the DAVID software that identified the complement and coagulation pathways as specifically enriched in the IUGR kidney. As an example, the intrinsic prothrombin pathway is shown (Figure 7); numerous coagulation factors are consistently up-regulated, demonstrating a severe alteration of this pro-coagulation cascade in the IUGR kidney, and suggesting an increased risk of thrombosis and clotting. The induction levels ranged from 2.2 for COL genes to ~6-7 fold for Kallikrein 1 and FGA.

**4.4 In the liver**

In this organ, three connected signaling pathways were identified as enriched: PKA signaling, Adenylate Cyclase activation signaling and Negative regulation of Galpha I GDP-GTP exchange signaling), with similar p values ranging from 0.0012 to 0.0017. The adenylate cyclase signaling pathway is represented in Supplementary Figure 4A. GPCR transcripts are overall induced, but this induction is very different according to the genes analyzed. Dopamine receptors are induced from 2.4 to 5.6 fold, Glutamate receptors GRM5 and GRM4 are induced 2.98 and 10.05 fold respectively. By contrast angiotensin receptors and adenosine receptor A3 are decreased 3.3 to 3.7 fold. In addition, RGS (Regulator of G protein genes) are overall repressed in the liver. G protein cascades are important regulators of cell metabolism, that contribute to regulate systemic functions such as embryonic development, learning and memory, and homeostasis . In the case of the liver, the down-regulation can be interpreted as a slowing down of the liver cell machinery in IUGR. One interpretation would be that systems that are not strictly indispensable are turned off in the liver (may be associated to its detoxification function) when there is a shortage in nutrients. The same type of down-regulation is exemplified by the Heteromeric GPCR signaling pathway in the liver (Supplementary Figure 4B).

**4.5 In the lungs**

In the lungs, 9 pathways were statistically significant (Table 4B). Very interestingly, the most significant were the EPO signaling and the IL7 signaling pathways (Supplementary Figure 5A and 5B). While the EPO (AKT-BAD signaling) was enriched in genes either induced or repressed, the IL7 signaling was systematically down-regulated. IL7 inactivates Bad signaling and is anti-apoptotic . The survival effects of interleukin-7 is mediated through Janus kinase 3 (JAK3), activated JAKs then activate signal transducers and activators of transcription (STATs) . The systematic inactivation of genes in this pathway suggests an overall lessened resistance to apoptosis in the lungs. The EPO pathway which also activates JAK signaling is preserved in the IUGR lungs. It suggests that there is a ‘choice’ between two survival pathways in case of protein shortage.
